# Transcriptome profiling of subepithelial PDGFRα cells in colonic mucosa reveals several cell-selective markers

**DOI:** 10.1371/journal.pone.0261743

**Published:** 2022-05-13

**Authors:** Se Eun Ha, Byungchang Jin, Brian G. Jorgensen, Hannah Zogg, Lai Wei, Rajan Singh, Chanjae Park, Masaaki Kurahashi, Sei Kim, Gain Baek, Sandra M. Poudrier, Moon Young Lee, Kenton M. Sanders, Seungil Ro

**Affiliations:** 1 Department of Physiology and Cell Biology, University of Nevada School of Medicine, Reno, Nevada, United States of America; 2 Department of Internal Medicine, Division of Gastroenterology and Hepatology, University of Lowa, Lowa City, Lowa, United States of America; 3 Department of Physiology, Wonkwang Digestive Disease Research Institute and Institute of Wonkwang Medical Science, School of Medicine, Wonkwang University, Iksan, Chonbuk, Korea; Duke University, UNITED STATES

## Abstract

Subepithelial platelet-derived growth factor receptor alpha (PDGFRα)^+^ cells found in the colonic mucosal tissue come in close contact with epithelial cells, immune cells, neurons, capillaries, and lymphatic networks. Mucosal subepithelial PDGFRα^+^ cells (MuPαC) are important regulators in various intestinal diseases including fibrosis and inflammation. However, the transcriptome of MuPαC has not yet been elucidated. Using Pdgfra-eGFP mice and flow cytometry, we isolated colonic MuPαC and obtained their transcriptome data. In analyzing the transcriptome, we identified three novel, and selectively expressed, markers (*Adamdec1*, *Fin1*, and *Col6a4*) found in MuPαC. In addition, we identified a unique set of MuPαC-enriched genetic signatures including groups of growth factors, transcription factors, gap junction proteins, extracellular proteins, receptors, cytokines, protein kinases, phosphatases, and peptidases. These selective groups of genetic signatures are linked to the unique cellular identity and function of MuPαC. Furthermore, we have added this MuPαC transcriptome data to our Smooth Muscle Genome Browser that contains the transcriptome data of jejunal and colonic smooth muscle cells (SMC), interstitial cells of Cajal (ICC), and smooth muscle resident PDGFRα^+^ cells: (https://med.unr.edu/physio/transcriptome). This online resource provides a comprehensive reference of all currently known genetic transcripts expressed in primary MuPαC in the colon along with smooth muscle resident PDGFRα cells, SMC, and ICC in the murine colon and jejunum.

## Introduction

Ingested food, through various chemical and mechanical signaling pathways, induces peristaltic reflexes in the gut. Due to this motility, cells present in intestinal villi and colonic mucosa are responsive to both chemosensory and mechanosensory signaling [1]. Mucosal subepithelial PDGRFα^+^ cells (MuPαC, aka subepithelial fibroblasts or fibroblast-like cells), located in the basement membrane under the epithelial layer of the colon, participate in the creation of contractile cellular networks via gap junctions [1]. These cells form subepithelial reticular intertwined networks around the crypts [2]. The networks enclose the lamina propria, in which MuPαC are in close proximity to neural and capillary networks, as well as myofibroblasts, epithelium, and immune cells [1].

MuPαC are closely associated with, but distinct from, myofibroblasts that express both α-smooth muscle actin (aka α-SMA: *Acta2*) and smooth muscle myosin (*Myh11*) [3]. Both MuPαC and myofibroblasts in the lamina propria are mesenchymal cells that predominantly originate from the visceral mesoderm [4]. Together, MuPαC and myofibroblasts play a role in acute and chronic epithelial injury, fibrosis, chronic inflammatory diseases, and colitis-associated cancer [2].

Previously, our group has reported that primary MuPαC isolated from colonic mucosa express Toll-like receptor genes, purinergic receptor genes, 5-hydroxytryptamine (5-HT) 4 receptor gene, and hedgehog signaling genes [3]. However, a comprehensive resource that encompasses genome-wide transcriptomic analysis within these cells still has yet to be developed.

We have previously isolated GFP-labeled PDGRFα cells from the jejunal and colonic muscularis of Pdgfra-eGFP mice [5], and characterized genome-scale gene expression data from these cells. With this trove of data, our group constructed a Smooth Muscle Genome Browser [6] linked to the bioinformatics data repository found at the University of California, Santa Cruz (UCSC) genome database [7]. For this study, we utilized a similar strategy to isolate MuPαC from Pdgfra-eGFP mice and then sequenced the transcriptomes of these cells, as well as whole mucosal tissue from the murine colon. This information was incorporated into the previously mentioned UCSC Smooth Muscle Genome Browser. Through analysis of the obtained transcriptome, we were able to identify several new cell-selective markers for MuPαC including the metalloendopeptidase ADAM-Like Decysin 1 (*Adamdec1*), fibronectin 1 (*Fin1*), and collagen type VI alpha 4 (*Col6a4*). We also identified several gene categories expressed in MuPαC including those encoding for growth factors, transcription factors, receptors, gap junction proteins, extracellular proteins, cytokines, peptidases, kinases, and phosphatases that are characteristic of MuPαC cellular identity and function. The MuPαC transcriptome we have added to the UCSC Smooth Muscle Genome Browser will serve as a resource that provides vital information about possible cellular structure, variously expressed transcript isoforms, and further insights into the regulation of all genes expressed in these cells.

## Methods and materials

### Animal and tissue preparation

*Pdgfra*^*eGFP/+*^ mice were obtained from Jackson Laboratory [8]. Mice were housed 4 per cage, maintained on a 12–12 hour light-dark cycle, and given access to food and water. All experiments were performed using 4–8 week old male and female *Pdgfra*^*eGFP/+*^ mice. The animal protocol was approved by the Institutional Animal Care and Use Committee at the University of Nevada-Reno Animal Resources. All experiments were performed in accordance with institutional guidelines and regulations.

### Flow cytometry and fluorescence-activated cell sorting (FACS)

Cells were dispersed from the colonic mucosa of *Pdgfra*^*eGFP/+*^ mice using an enzyme digestion buffer comprised of 4mg/ml collagenase type 2, 8mg/ml trypsin inhibitor, 8mg/ml bovine serum albumin and 0.125mg/ml papain and incubated at 37°C for 30 min. GFP^+^ PDGRFα cells were sorted from dispersed cells using FACS [5]. Isolated GFP^+^ PDGRFα^high^ cells (as differenctiated PDGFRα cells) from *Pdgfra*^*eGFP/+*^ mice (15 males and 15 females) were lysed and these cell lysates were pooled together with all other lysate samples. This pooled lysate from thirty *Pdgfra*^*eGFP/+*^ mice was used as one collective sample in the isolation of total RNAs.

### Isolation of total RNAs

Total RNA was isolated from the colonic mucosa of mice using the mirVana miRNA isolation kit (Life Technologies, Carlsbad, CA) according to the manufacturer’s instructions. The quality of total RNAs was analyzed using a NanoDrop 2000 Spectrometer (Thermo Fisher Scientific, Waltham, MA) and a 2100 Bioanalyzer (Agilent Technologies, Santa Clara, CA).

### Construction of RNA-seq libraries and next-generation sequencing

Two RNA-seq libraries were generated and sequenced via Illumina HiSeq 2000 (Illumina, San Diego, CA) following the vendor’s instruction at LC Sciences (Houston, TX) as previously described [5].

### Bioinformatics data analysis

Paired-end sequencing reads were processed and analyzed as previously described [9]. A cutoff of FPKM = 0.025 generated equal false positive and false negative ratios of reliability. The expression level of transcripts with a FPKM value of less than 0.025 were considered to be 0.

### Real time polymerase chain reaction

cDNA libraries were made using reverse transcription of the total RNAs isolated from FACS-purified MuPαC (n = 6) from colonic mucosa and smooth muscle PDGFRα cells (SMPαC: n = 6) [5], Interstitial cells of Cajal (ICC: n = 6) [10] and smoothe muscle cells (SMC: n = 6) [11] from colonic muscularis as well as colonic mucosa (n = 5) and smooth muscle, (n = 6) as previously described [5, 10, 11] [n = 5–6 mice (3 males and 2/3 females) per cell and tissue type]. Reverse-transcription polymerase chain reaction (RT-PCR) and quantitative PCR (qPCR) analyses of cDNA were performed as previously described [5]. All primer sequences used can be found in S1 Table.

### Confocal microscopy and immunohistochemical analysis

Frozen murine tissues were fixed in cold acetone and 4% PFA before 8 μm cryosections were cut using a cryostat. Cryosections were then placed onto slides coated with Vectabond. anti-PDGFR-alpha (goat, 1:100, R&D system, MN), anti-Fibronectin (FN1) (rabbit, 1:100, abcam, MA), anti-Collagen VI (COL6) (rabbit, 1:200, abcam, MA), anti-PLAU (rabbit, 1:100, abcam, MA), anti-PROCR (Rabbit, 1:50, Bioss antibodies, MA), anti-BMP7 (Rabbit, 1:100, AVIVA system biology, CA), anti-SEMA3F (Rabbit, 1:50, Bioss antibodies, MA), and anti-PCSK6 (Goat, 1:100, antibodies-online, GA) were the primary antibodies used. Primary and secondary antibodies were diluted in 4% skim milk/1x TBS/0.1% Triton-X114. Each slide was washed twice with 1x TBS and treated with Fluoroshield mounting medium with DAPI (Abcam, ab104139) after incubation with the secondary antibodies. An Olympus FV1000 confocal laser scanning microscope (Olympus, Tokyo, Japan) was used to capture the immunohistochemically stained images and these images were analyzed through Fluoview FV10-ASW 3.1 Viewer software (Olympus, Tokyo, Japan).

### Statistical analysis

qPCR data obtained in the present study was compared using a one-way analysis of variance (ANOVA) in order to determine whether the differences were statistically significant. Measured variables were expressed as the mean ± standard errors of the mean (SEM). The differences in mean values between the two groups (MuPaC and SMPaC) were evaluated and considered significantly different when *P < 0.05 and **P < 0.*01*.

## Results

### Identification and isolation of mucosal subepithelial PDGFRα^+^ cells

**Fig 1:**
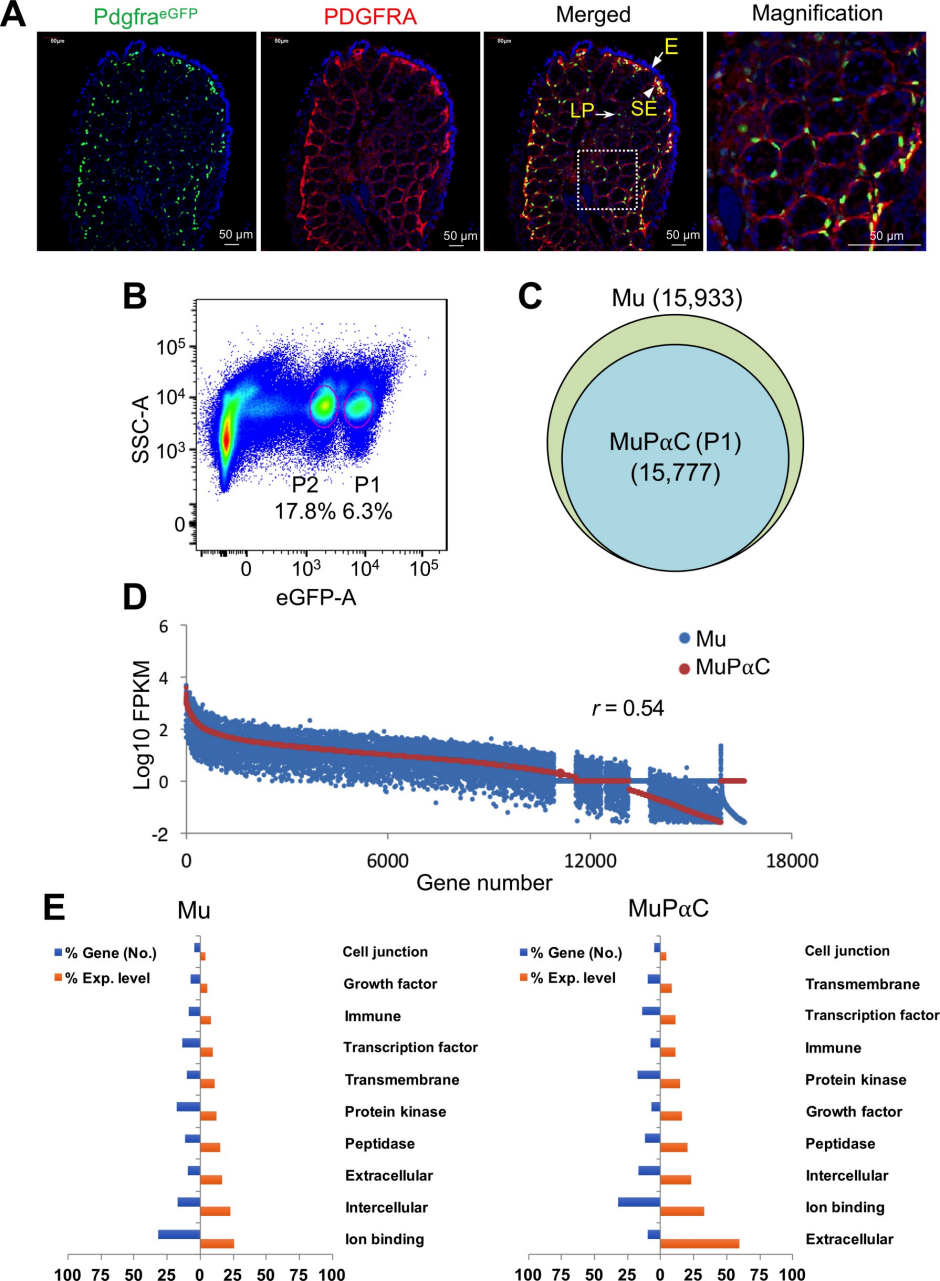
Identification of colonic mucosal subepithelial PDGFRα^+^ (MuPαC) cells and analysis of their transcriptome. *A*: PDGFRα^+^ cells in the colonic mucosa identified with Pdgfra-eGFP and through PDGFRA antibody. Pdgfra-eGFP mice express the H2B-eGFP (nuclear eGFP) fusion gene from the *Pdgfra* locus. L, lumen; SE, subepithelium; E, epithelium; LP, lamina propria. *B*: Primary eGFP^+^ PDGFRα^+^ populations (bright, P1, MuPαC, and dim, P2, MuPαC) from colonic mucosa identified (circled) through flow cytometry. *C*: Chart showing the number of genes identified in colonic mucosal tissue (Mu) and MuPαC cells in the colonic mucosa through RNA-seq. *D*: Comparison of expression levels of genes in Mu and MuPαC. *E*: Gene ontologies reported in Mu and MuPαC. The gene ontology (GO: function, process, and component) for Mu-/ MuPαC-specific genes was analyzed, and key GO terms were compared using normalized expression (FPKM) percentile. Blue and orange bars indicate a percentage of the gene number and an expressed amount of the gene, in each GO term category, to a total gene number or expressed amount respectively.

Mucosal subepithelial PDGFRα^+^ cells (MuPαC) were identified through eGFP expression within the subepithelial region of colonic mucosa in Pdgfra^eGFP/+^ mice [8] (Fig 1A). The identity of MuPαC was confirmed through immunohistochemical labeling with anti-PDGFRA antibodies that coincided with endogenous nuclear eGFP, as seen in previous work [3]. The PDGFRA protein is mainly localized in the plasma membrane of MuPαC, while eGFP is exclusively found within the nucleus of the cells as the eGFP gene is fused with a human histone H2B type 2-E gene in Pdgfra^eGFP/+^ mice [8]. MuPαC are in close proximity to each other under the epithelial barrier (Fig 1A). MuPαC are concentrated at the subepithelial area of the cryptic plateau within plicae in contrast to the lower number seen in the cryptic base and axis. Primary MuPαC from colonic mucosa were further analyzed through the use of flow cytometry. Our group previously reported two distinct populations of eGFP^+^ MuPαC within the murine colonic mucosa: cells with brighter eGFP fluorescence (report higher expression of PDGFRα: P1) and cells with dimmer eGFP fluorescence (report lower expression of PDGFRα: P2) [3]. The P1 and P2 cells within eGFP^+^ MuPαC, identified by fluorescence-activated cell sorting (FACS), were 6.3% and 17.8%, respectively, of the total events (Fig 1B), which was consistent with our previous cell sorting data [3]. Since P1 cells express *Pdgfra* at a higher level than P2 cells [3], we identified and isolated only the brighter eGFP^+^ MuPαC population (MuPαC, P1) for RNA-seq. We sorted MuPαC from 30 mice (15 from each sex), extracted total mRNAs from each mouse’s isolated colonic mucosa, and pooled these mRNA samples together. This pooling process was also carried out on unsorted cells (colonic mucosal tissue).

### Transcriptomic analysis of mucosal subepithelial PDGFRα^+^ cells

To identify the genes expressed within MuPαC, we obtained and analyzed the transcriptomes of isolated mucosal tissue (Mu) and MuPαC. The transcriptomes consisted of 15,933 (Mu) and 15,777 (MuPαC) known genes (Fig 1C and Table 1). We obtained 169 and 154 million reads, of which 91% and 92% were mapped to the genome in Mu and MuPαC, respectively. We found 52,113 and 51,282 unique gene isoforms in Mu and MuPαC, respectively. Complete lists of all isoforms identified in this study along with tracking IDs, gene ID/names, chromosome location, isoform length, and expression levels in both Mu and MuPαC can be found in Table 1. MuPαC expressed an average of 3 isoforms per gene that were produced from alternative transcription start sites, and/or alternative splicing sites (NCBI GEO GSM1388414 and GSM1388415, S2 Table). Most genes (15,777) were expressed in both Mu and MuPαC; however, we found 156 genes that were expressed in Mu that were not found to be expressed in MuPαC (Fig 1C). A complete list of the genes expressed in Mu and MuPαC with their combined isoform expression levels and numbers of splice variants can be found in S3 Table. Although most genes are expressed in both Mu and MuPαC, the overall expression profiles of both samples were not very similar (correlation coefficient = 0.54) (Fig 1D). To further investigate cellular identity and function from our transcriptome data, we employed gene ontology (GO) analysis of genes abundantly expressed in Mu and MuPαC. Key GO terms and numbers of genes found to be associated with each term obtained from both samples were similar. The most highly expressed genes in the Mu population are involved in ion binding. In contrast, genes coding for extracellular proteins were the most highly expressed category in MuPαC (Fig 1E). This suggests that MuPαC may have an important role in extracellular function.

**Table 1:** Summary of transcriptomes obtained from colonic mucosal tissue (Mu) and subepithelial PDGFRα^+^ cells (MuPαC).

Sample	Total read	Mapped read	Known gene	Total isoform	Average isoform
**Mu**	168,835,236	153,208,218	15,933	52,113	3.3
**MuPαC**	154,151,508	141,592,530	15,777	51,282	3.3

### Identification of genes exclusively expressed in mucosal subepithelial PDGFRα cells

**Fig 2:**
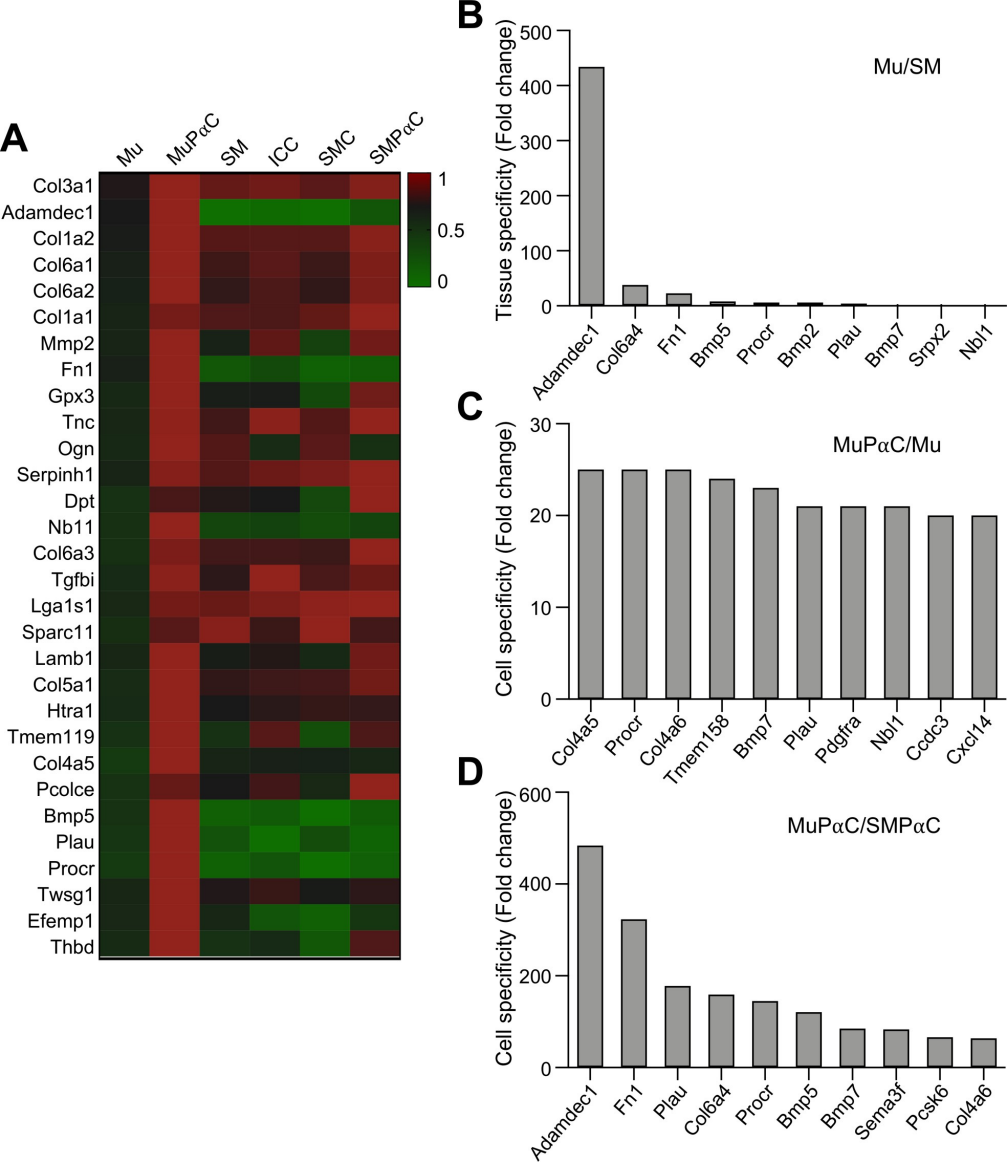
Identification of genes expressed in isolated colonic MuPαC. *A*: A heat map of genes expressed in colonic MuPαC compared to colonic mucosa (Mu), colonic smooth muscle (SM), interstitial cells of Cajal (ICC), smooth muscle cells (SMC), and smooth muscle PDGFRα^+^ cells (SMPαC). *Col3a1* and Adamdec1 are highly expressed in colonic MuPαC. *B*: Colonic Mu-specific genes compared to colonic SM. *C*: MuPαC-specific genes compared to Mu. *D*: Colonic MuPαC-specific genes compared to colonic SMPαC.

**Fig 3:**
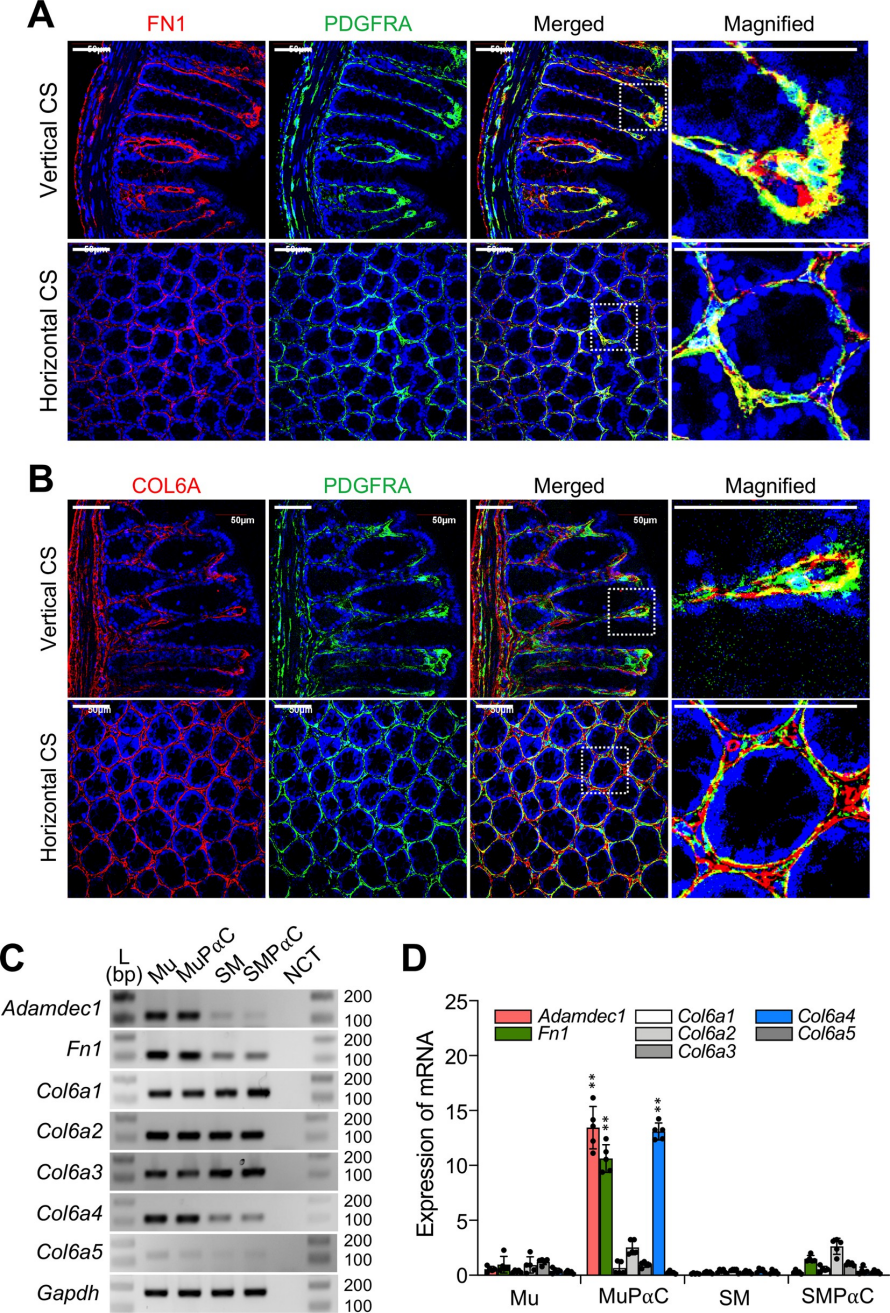
Selective expression of FN1 and COL6A4 in colonic MuPαC. *A* and *B*: Restricted expression of FN1 and COL6A protein within colonic subepithelial PDGFRA^+^ cells. Anti-FN1 and anti-COL6A antibodies were used. Vertical and horizontal cross-sections (CS) images are indicated. Scale bars are 50 μm. *C*: Expression of *Adamdec1*, *Fn1*, and collagen type 6 isoforms (*Col6a1*-*5*) in Mu, isolated MuPαC, SM, and isolated SMPαC examined by RT-PCR. *D*: Quantitative analysis of *Adamdec1*, *Fn1*, and *Col6a1*-*5* mRNA expression in Mu (n = 5), isolated MuPαC (n = 5), SM (n = 5), and isolated SMPαC (n = 6) measured by qPCR. ** p ≤ 0.01, MuPαC versus SMPαC. *Gapdh* was used as an endogenous control.

We have previously obtained and analyzed the transcriptomes of colonic smooth muscle tissue (SM) as well as three cell types that reside within gastrointestinal tissue: smooth muscle cells (SMC) [9], interstitial cells of Cajal (ICC) [10], and smooth muscle PDGFRα^+^ cells (SMPαC) [5]. To identify genes selectively expressed in mucosal subepithelial PDGFRα^+^ cells (MuPαC), we analyzed and compared the transcriptomes of MuPαC and Mu to the existing transcriptomes of SM, SMC, ICC, and SMPαC. We identified 76 genes that are highly, and selectively, expressed within colonic MuPαC when compared to Mu, SM, and the previously mentioned cell types (SMC, ICC, and SMPαC) (S4 Table). The thirty most selectively enriched gene expression signatures in MuPαC are shown in Fig 2A. The top three most highly enriched genes in MuPαC are *Col3a1*, *Adamdec1* and *Col1a2*. *Adamdec1*, *Fn1*, and *Col6a4* also show selective expression in Mu compared to SM (Fig 2B). Additionally, the top three MuPαC-enriched genes in comparison to Mu are *Procr*, *Col6a4* and *Bmp7* (Fig 2C). Lastly, the top three MuPαC-enriched genes vs SMPαC are *Adamdec1* and *Fn1*, and *Plau* (Fig 2D). Taken together, the most selective genes in MuPαC at mRNA levels include *Adamdec1*, *Fn1*, and *Col6a4*. To validate the cell-restricted expression of genes expressed in MuPαC, we selected 8 genes (*Adamdec1*, *Fn1*, *Col6a4*, *Plau*, *Procr*, *Bmp7*, *Sema3f*, and *Pcsk6*) and performed immunohistochemistry on murine colonic tissue in order to label the protein product of each previously listed gene (Figs 3A and 3B and S1). This screening identified ADAMDEC1, FN1, and COL6A4 as being selectively expressed in MuPαC. In a separate parallel study, we found that ADAMDEC1 is not only a selective marker for MuPαC but also a biomarker induced by colonic mucosal inflammation (in review) [12]. However, PLAU, PROCR, BMP7, SEMA3F, and PCSK6 expression were not able to efficiently label MuPαC (S1 Fig). Restricted localization of FN1 and COL6A isoforms in MuPαC isolated from colonic mucosa is shown in Fig 3. FN1 was more prominently found in MuPαC compared to SMPαC (Fig 3A). In addition, the FN1 protein was abundantly detected in mesothelial cells in the serosal layer. FN1 abundantly colocalized in subepithelial PDGFRα^+^ cells under the epithelial cells in cryptic plateaus (vertical sections) and cryptic bases or axes (horizontal sections), where epithelial stem/progenitor cells are located. Another marker, COL6A4, was labeled with the anti-collagen 6 (COL6A) antibody due to the isoform specific anitbody (anti-COL6A4) being unavailable. COL6A showed about equal signal strength in MuPαC as FN1. However, there are five collagen type 6 genes, *Col6a1-5*, expressed in the colonic mucosa of mice (S3 Table). This results in the labeling of all proteins that are translated from the collagen type 6 genes when using the COL6A antibody. Our transcriptome data show that MuPαC have medium to high expression of *Col6a1-4*, while these cells have very low expression of *Col6a5* (S3 Table). Thus, the signal in MuPαC is likely mostly from COL6A1-4. Additionally, this antibody would likely also label SMPαC as these cells have abundant expression of three of the COL6A isoforms (S4 Table).

However, according to the transcriptome data, *Col6a4* is expressed at very low levels in SM and SMPαC (Fig 2A and S4 Table). Similar to FN1, COL6A isoforms were also found in subepithelial PDGFRα^+^ cells at the cryptic plateaus and axes or bases (Fig 3B). To further validate expression of *Fn1* and *Col6a4* in MuPαC, we examined their cell-restricted expression in colonic MuPαC and SMPαC by RT-PCR. Consistent with the transcriptome data, colonic mucosa tissue and isolated MuPαC detected varying transcript levels of *Adamdec1*, *Fn1*, *Col6a1*, *Col6a2*, *Col6a3*, and *Col6a4* and very low expression of *Col6a5* across all samples (Fig 3C). In addition, colonic SM tissue and isolated SMPαC showed very low transcript levels of *Adamdec1*, *Fn1*, and *Col6a4* and abundant transcript levels of *Col6a1*, *Col6a2*, and *Col6a3*. In terms of differences in expression levels between MuPαC and SMPαC, we observed *Adamdec1*, *Col6a4*, and *Fn1* had significantly higher expression in MuPαC than SMPαC through qPCR analysis (Fig 3D). These findings were consistent with our transcriptome data (S4 Table). Taken together, the immunohistochemical, RT-PCR and qPCR data show that *Adamdec1*, *Fn1*, and *Col6a4* are likely selective markers for MuPαC.

### Identification of growth factors, transcription factors, cell signaling genes, receptors and receptor binding proteins expressed in mucosal subepithelial PDGFRα cells

**Fig 4:**
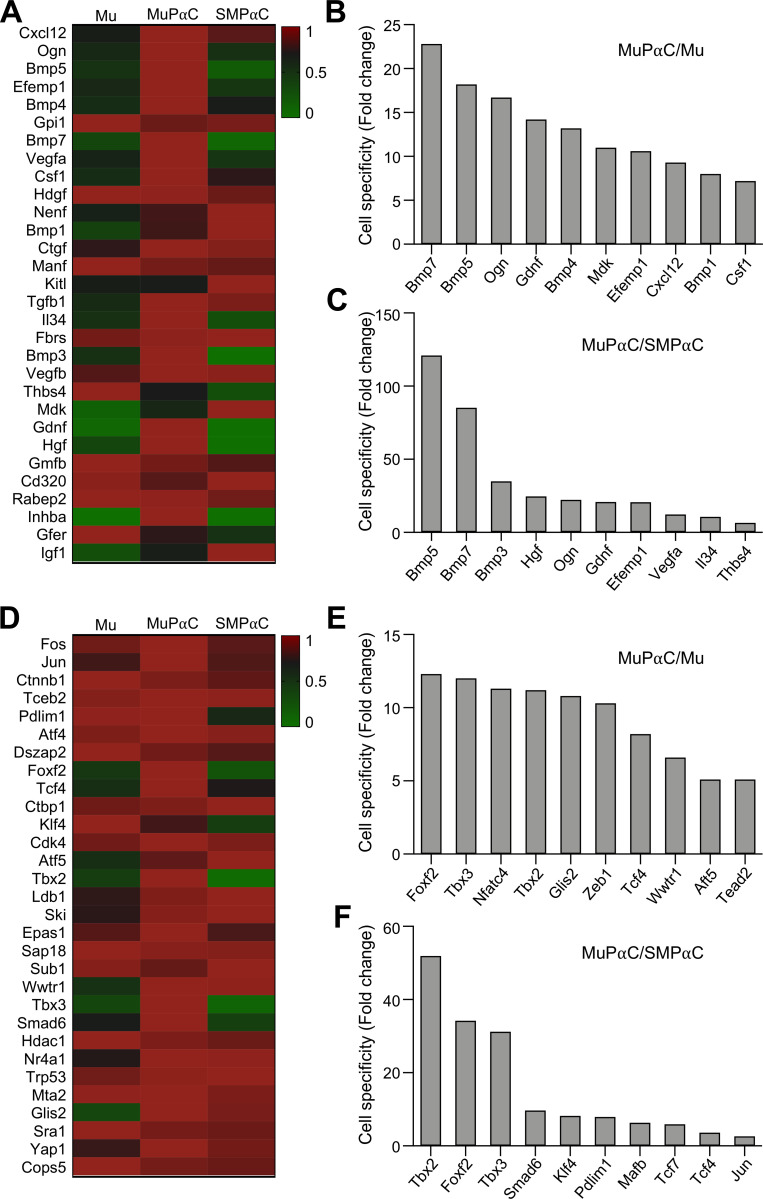
Identification of the growth factors and transcription factors predominantly expressed in colonic MuPαC. *A*: A heat map of the growth factors enriched in MuPαC compared to mucosal tissue (Mu) and smooth muscle PDGFRα^+^ cells (SMPαC). *B*: MuPαC-specific growth factors compared to Mu. *C*: MuPαC-specific growth factors compared to SMPαC. Sorted by MuPαC/Mu, cut off 10 fold MuPαC in MuPαC/Mu; sorted by MuPαC/SMPαC, cut off 10 fold MuPαC and 0 fold SMPαC in MuPαC/SMPαC. *D*: A heat map of the transcription factors enriched in MuPαC compared to Mu and SMPαC. *E*: MuPαC-specific transcription factors compared to Mu. *F*: MuPαC-specific transcription factors compared to SMPαC. Sorted by MuPαC/Mu, cut off 10 fold MuPαC in MuPαC/Mu; sorted by MuPαC/SMPαC, cut off 10 fold MuPαC and 0 fold SMPαC in MuPαC/SMPαC.

MuPαC expressed 52 growth factors (S5 Table). The thirty most predominantly expressed growth factors in MuPαC, compared to Mu, are shown in Fig 4A. *Cxcl12* and *Ogn* appeared to be the most highly expressed, while *Bmp7* and *Bmp5* were the most specific to MuPαC (Fig 4B). All ten of the most predominantly expressed growth factors in MuPαC were also expressed in SMPαC with *Gpi1* being the only growth factor of these ten that is more highly expressed in SMPαC than levels seen in MuPαC (Fig 4B). *Bmp5* and *Bmp7* had very low expression in SMPαC but high expression in MuPαC (Fig 4C), suggesting that these two growth factors may be required for the growth of MuPαC.

In addition, MuPαC expressed 134 transcription factors (S6 Table). *Fos* and *Jun* were the most highly expressed transcription factors in MuPαC (Fig 4D), with these two genes also having highly expressed in SMPαC (Fig 4D). *Tbx2* and *Foxf2* appeared to be the most specific to MuPαC over Mu (Fig 4E), while *Tbx2* and *Foxf2* were the most specific to MuPαC over SMPαC (Fig 4F).

**Fig 5:**
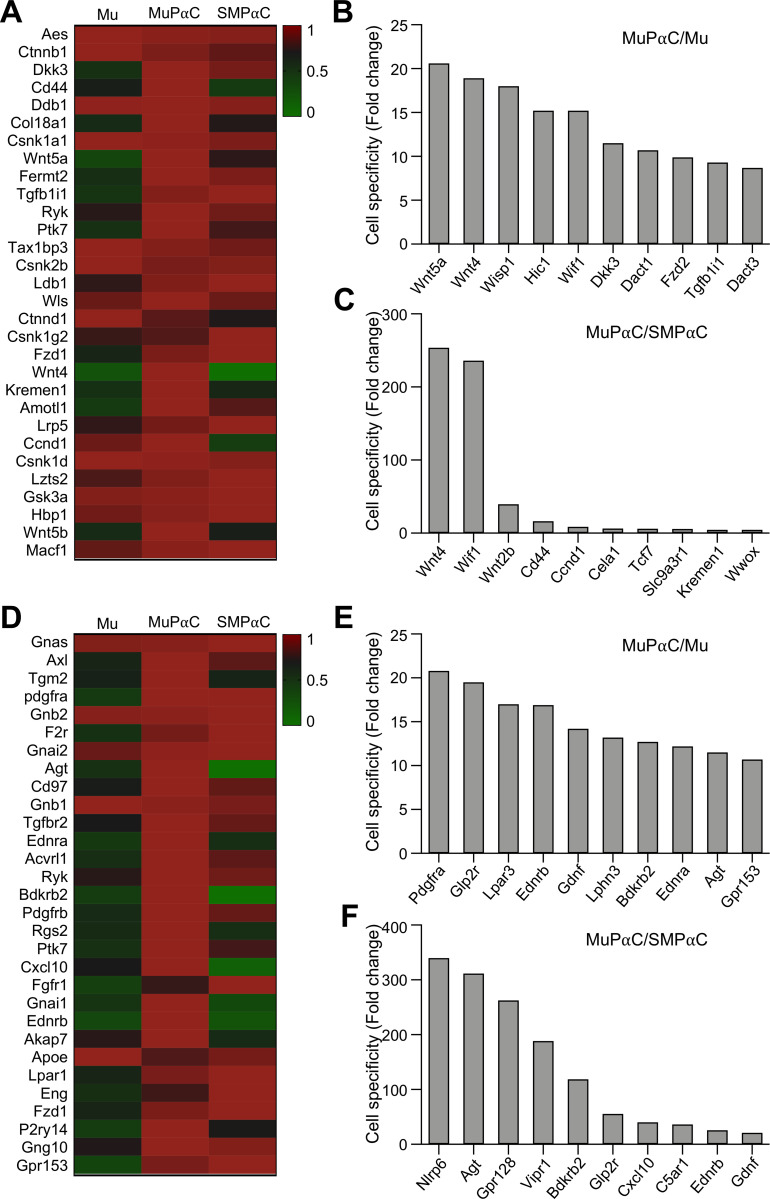
Identification of the cell signaling genes, receptors and receptor binding proteins predominantly expressed in colonic MuPαC. *A*: A heat map of the cell signaling genes enriched in MuPαC compared to mucosal tissue (Mu) and smooth muscle PDGFRα^+^ cells (SMPαC). *B*: MuPαC-specific cell signaling genes compared to Mu. *C*: MuPαC-specific cell signaling genes compared to SMPαC. Sorted by MuPαC/Mu, cut off 10 fold MuPαC in MuPαC/Mu; sorted by MuPαC/SMPαC, cut off 10 fold MuPαC and 0 fold SMPαC in MuPαC/SMPαC. *D*: A heat map of the receptor binding proteins enriched in MuPαC compared to Mu and SMPαC. *E*: MuPαC-specific receptor binding proteins compared to Mu. *F*: MuPαC-specific receptor binding proteins compared to SMPαC. Sorted by MuPαC/Mu, cut off 10 fold MuPαC in MuPαC/Mu; sorted by MuPαC/SMPαC, cut off 10 fold MuPαC and 0 fold SMPαC in MuPαC/SMPαC.

MuPαC also expressed 133 genes involved with cell signaling (S7 Table). The thirty most predominantly expressed cell signaling genes in MuPαC are shown in Fig 5A. Each one of these cell signaling genes was also found to be expressed in SMPαC, albeit at differing levels of expression. Interestingly, two Wnt singling genes (*Wnt5a* and *Wnt4*) were specifically expressed in MuPαC when compared to either Mu (Fig 5B) or SMPαC (Fig 5C). *Wnt4* and *Wif1* were more highly specific to MuPαC as compared to levels found in SMPαC (Fig 5C).

Finally, MuPαC expressed 203 receptor and receptor binding protein genes (S8 Table). *Gnas* was the most highly expressed and *Pdgfra* was the most specifically expressed gene in MuPαC (Fig 5D and 5E). These two genes were also highly expressed in SMPαC (Fig 5D). *Nlrp6* and *Agt* were the most specific genes to MuPαC over SMPαC (Fig 5F). However, *Nlrp6* was also highly expressed in Mu, while *Agt* was expressed at much lower levels in Mu when compared to MuPαC levels (S8 Table). This suggests that *Nlrp6* may be expressed in other mucosal cells, while *Agt* was predominantly expressed in MuPαC.

### Identification of predominantly expressed genes related to gap junctions and extracellular activity in mucosal subepithelial PDGFRα cells

**Fig 6:**
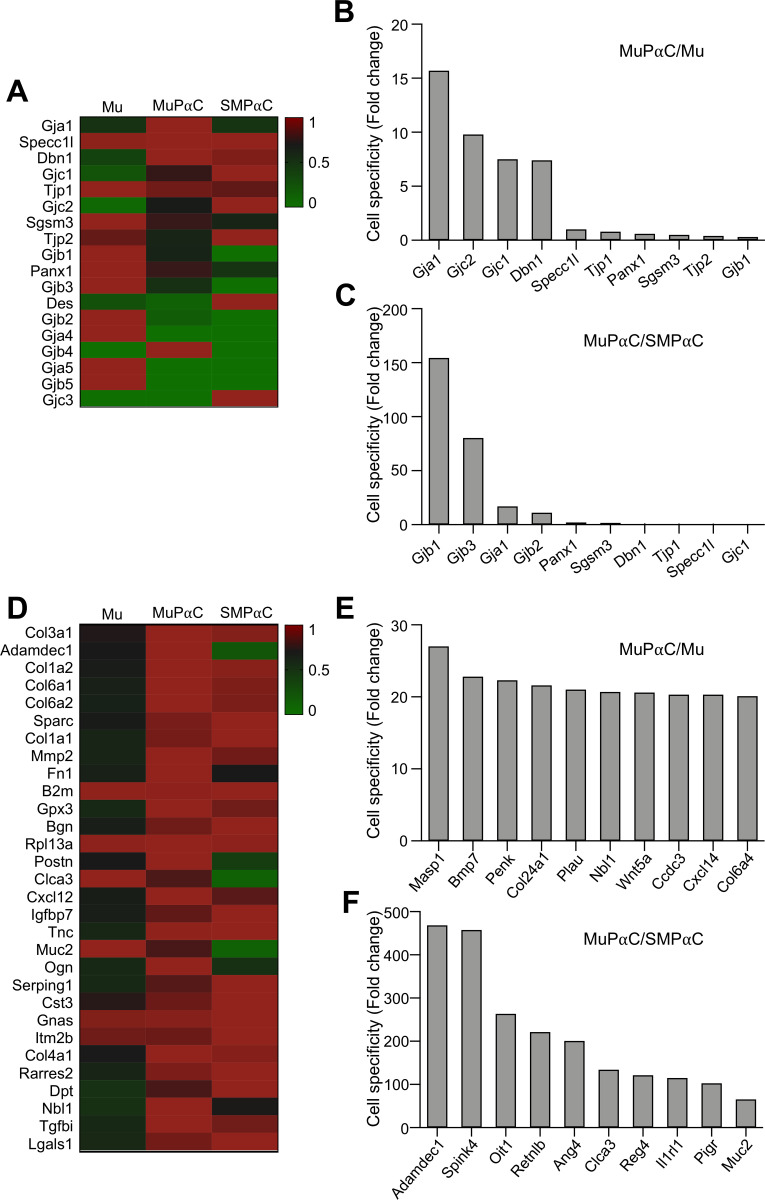
Identification of the gap junction and extracellular proteins predominantly expressed in colonic MuPαC. *A*: A heat map of the gap junction proteins enriched in MuPαC compared to mucosal tissue (Mu) and smooth muscle PDGFRα^+^ cells (SMPαC). *B*: MuPαC-specific gap junction proteins compared to Mu. *C*: MuPαC-specific gap junction proteins compared to SMPαC. *D*: A heat map of the extracellular proteins enriched in MuPαC compared to Mu and SMPαC. *E*: MuPαC-specific extracellular proteins compared to Mu. *F*: MuPαC-specific extracellular proteins compared to SMPαC. Sorted by MuPαC/Mu, cut off 10 fold MuPαC in MuPαC/Mu; sorted by MuPαC/SMPαC, cut off 10 fold MuPαC and 0 fold SMPαC in MuPαC/SMPαC.

MuPαC expressed 18 genes related to gap junctions (S9 Table). Of these genes, *Gja1* was the most highly and specifically expressed in MuPαC over both Mu and SMPαC (Fig 6A–6C). In SMPαC, *Des* was the most highly and specifically expressed (S9 Table). However, MuPαC specifically expressed Gjb1, Gjb3, and Gja1 over SMPαC (Fig 6C), suggesting that these gap junction proteins have a unique role in MuPαC.

MuPαC expressed 600 genes related to extracellular activity (S10 Table). *Col3a1* and *Adamdec1* were the most highly expressed extracellular activity genes in MuPαC (Fig 6D) with *Col3a1* being the most highly expressed in SMPαC, and *Adamdec1* being minimally expressed in SMPαC (Fig 6D). Many extracellular proteins, including *Masp1*, *Bmp7*, and *Penk*, are preferentially expressed in MuPαC over Mu (Fig 6E). Additionally, *Adamdec1* was the most specifically expressed gene in MuPαC over SMPαC (Fig 6F). Many extracellular activity genes are preferentially expressed in MuPαC over SMPαC: 44 genes are more than 100 fold enriched in MuPαC compared to SMPαC (S10 Table).

### Identification of cytokine, peptidase, protein kinase, and phosphatase genes found in mucosal subepithelial PDGFRα cells

**Fig 7:**
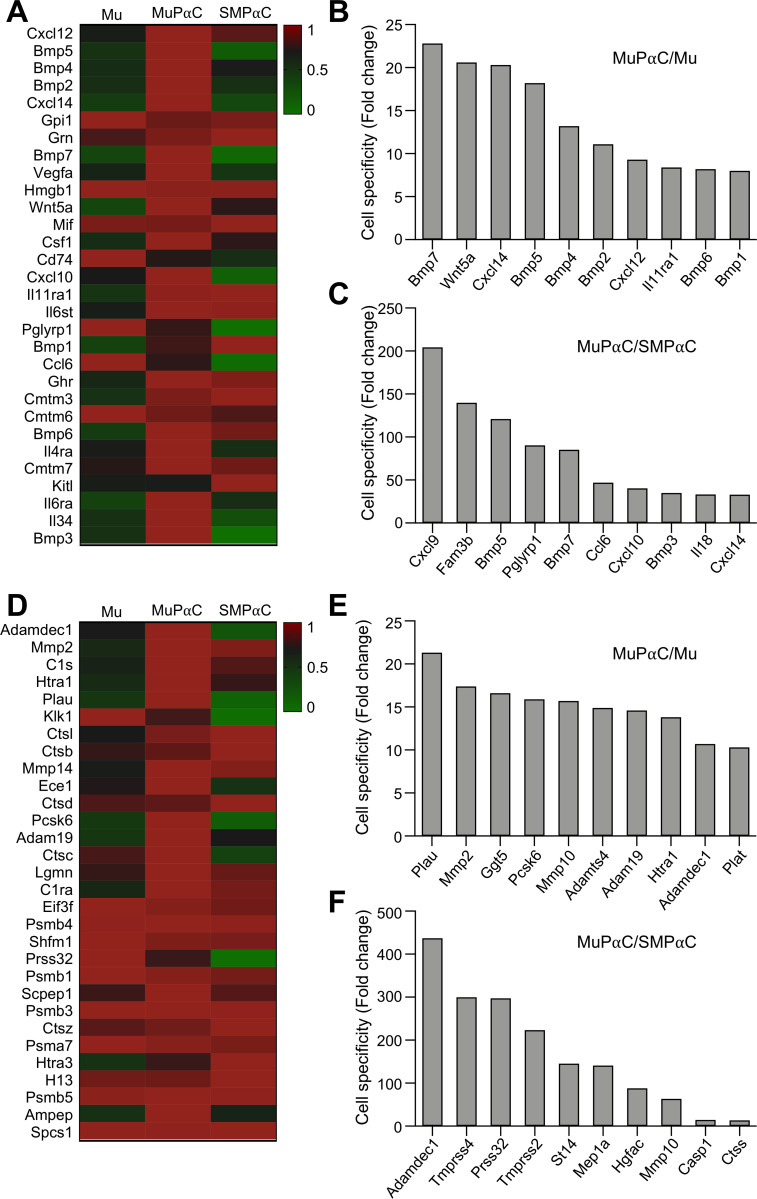
Identification of the cytokines and peptidases predominantly expressed in colonic MuPαC. *A*: A heat map of the cytokines enriched in MuPαC compared to mucosal tissue (Mu) and smooth muscle PDGFRα^+^ cells (SMPαC). Sorted by MuPαC/Mu, cut off 10 fold MuPαC in MuPaC/Mu; sorted by MuPαC/SMPαC cut off 10 fold MuPαC and 1 fold SMPαC in MuPαC/SMPαC. *B*: Cytokines enriched in MuPαC compared to colonic SMPαC. *C*: MuPαC-specific cytokines compared to SMPαC. *D*: A heat map of the peptidases enriched in MuPαC compared to Mu and SMPαC. *E*: MuPαC-specific peptidases compared to Mu. *F*: MuPαC-specific peptidases compared to SMPαC. Sorted by MuPαC/Mu, cut off 10 fold MuPαC in MuPaC/Mu; sorted by MuPαC/ SMPαC, cut off 10 fold MuPαC and 1 fold SMPαC in MuPαC/SMPαC.

MuPαC expressed 77 genes encoding for cytokines (S11 Table). The thirty most highly expressed genes encoding for cytokines within MuPαC are shown in Fig 7A. *Cxcl12* is the most highly expressed in MuPαC and SMPαC (Fig 7A). The genes most specific to MuPαC as compared to Mu are *Bmp7* and *Wnt5a* (Figs 7B and S1B and S1D). Twelve cytokine genes are preferentially expressed in MuPαC over SMPαC, with *Cxcl9* and *Fam3b* being the most specific (Fig 7C and S11 Table).

MuPαC expressed 283 peptidase genes (S12 Table). The thirty most highly expressed peptidases in MuPαC are shown in Fig 7D. *Adamdec1* (previously categorized as an extracellular gene in Fig 6D) is the most highly expressed peptidase in MuPαC, with a negligible expression level in SMPαC (Fig 7D and 7F). Interestingly many peptidase genes are preferentially expressed in MuPαC over SMPαC: 11 genes have an over 100 fold enrichment in MuPαC (S12 Table).

**Fig 8:**
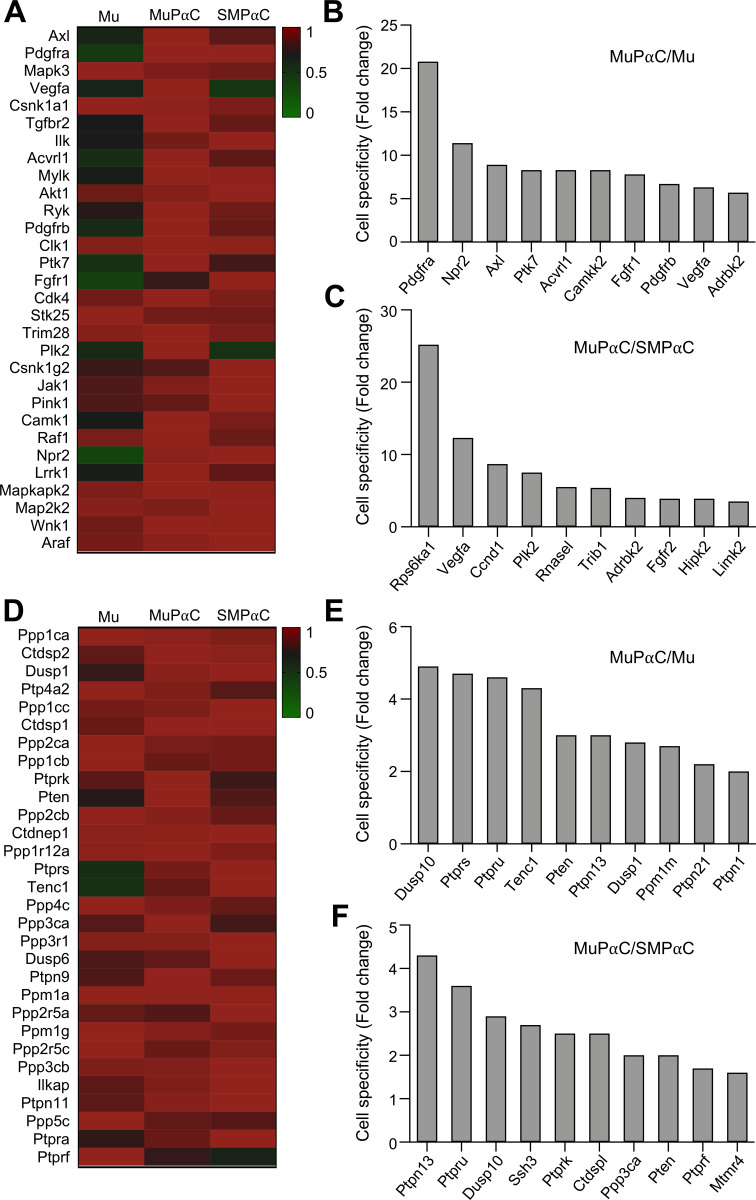
Identification of the protein kinases and phosphatases predominantly expressed in colonic MuPαC. *A*: A heat map of the protein kinases enriched in MuPαC compared to mucosal tissue (Mu) and smooth muscle PDGFRα^+^ cells (SMPαC). *B*: MuPαC-specific protein kinases compared to Mu. *C*: MuPαC-specific protein kinases compared to SMPαC. Sorted by MuPαC/Mu, cut off 10 fold MuPαC in MuPαC/Mu; sorted by MuPαC/SMPαC, cut off 10 fold MuPαC and 1 fold SMPαC in MuPαC/SMPαC. *D*: A heat map of the phosphatases enriched in MuPαC compared to Mu and SMPαC. *E*: MuPαC-specific phosphatases compared to Mu. *F*: MuPαC-specific protein phosphatases compared to SMPαC. Sorted by MuPαC/Mu, cut off 10 fold MuPαC in MuPαC/Mu; sorted by MuPαC/SMPαC, cut off 10 fold MuPαC and 1 fold SMPαC in MuPαC/SMPαC.

Finally, MuPαC expressed 354 protein kinase genes (S13 Table) and 105 phosphatase genes (S14 Table). The thirty most highly expressed protein kinases in MuPαC are shown in Fig 8A. *Axl* and *Pdgfra* (also previously categorized as a receptor in Fig 5D) were the most highly expressed kinases in MuPαC (Fig 8A). As expected, *Pdgfra* was also highly expressed in SMPαC, being the most specific to both PαC (Fig 8A and 8B). The two most specific genes to MuPαC as compared to SMPαC are *Rps6ka1* and *Vegfa* (Fig 8C). *Rps6ka1* was also highly expressed in Mu, but *Vegfa* was expressed at a much lower level in Mu, suggesting that *Rps6ka1* may be expressed in other mucosal cells; however, *Vegfa* is predominantly expressed in MuPαC (Fig 8A and S13 Table). The thirty most highly expressed phosphatase genes in MuPαC are shown in Fig 8D. Each one of these phosphatase genes were also abundantly expressed in SMPαC (Fig 8D). *Dusp10* and *Ptpn13* were the most specifically expressed in MuPαC over Mu and SMPαC, respectively (Fig 8E and 8F).

### Validation of MuPaC-selective genes

As shown in Figs 2 and 4–8, the 26 MuPαC-selective genes (*Col3a1*, *Adamdec1*, *Bmp7*, *Bmp5*, *Ogn*, *Foxf2*, *Tbx3*, *Tbx2*, *Wnt4*, *Wnt5a*, *Pdgfra*, *Nlrp6*, *Agt*, *Gja1*, *Gjb1*, *Gjb3*, *Dmp1*, *Cxcl9*. *Fam3b*, *Masp1*, *Penk1*, *Axl*, *Rps8ka1*, *Vegfa*, *Dusp10 and Ptpn13*) were indentified by the transcriptome analyses. To valiate the RNA-seq profiles of these genes, we quantified expression levels of each gene in isolated MuPαC, SMPαC, ICC, SMC, colonic Mu and SM tissue using qPCR analysis. Expression levels of the 24 genes were significantly higher in MuPαC than the other cell and tissue types, suggesting these genes are indeed MuPαC-selective (S2 and S3 Figs). The other two genes, *Pdgfra* and *Penk1*, were also more highly expressed in both MuPαC and SMPαC than SMC and ICC, implying they are MuPαC- and SMPαC-selective. These qPCR data confirmed the expression profiles of the 26 MuPαC-selective genes identified by the transcriptome analyses.

### Addition to UCSC Smooth Muscle Genome Browser

**Fig 9:**
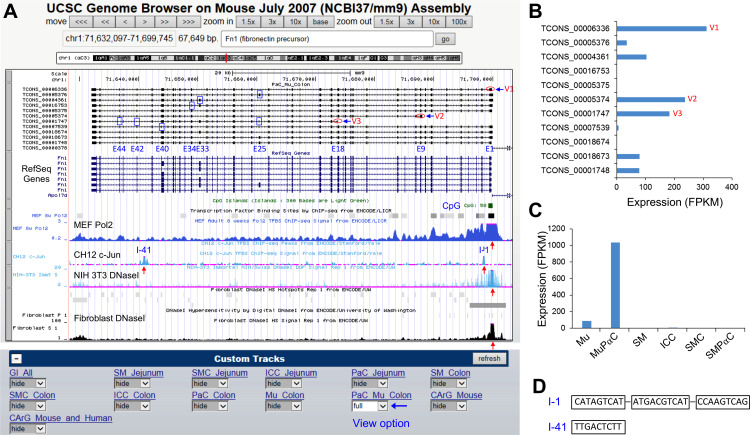
Genomic structure and expression data of *Fn1* analyzed using the UCSC Smooth Muscle Genome Browser. *A*: A genomic map view of *Fn1* variants expressed in MuPαC. Three alternative initial exons (V1-3) are circled and six alternative exons are boxed in blue. A CpG island denoted by a green box. Red arrows indicate either binding sites of Polymerase 2 (Pol2) in mouse embryonic fibroblasts (MEF), a heterodimer of FOS and JUN (c-Jun) constituting transcription factor AP1 in CH12, or DNase I hypersensitive sites in NIH 3T3 and fibroblasts. Custom Tracks have view options (hide, dense, squish, pack, full: full is selected in the image) for the transcriptome data of MuPαC (eGFP^+^-PaC Mu Colon). *B*: Expression (FPKM) levels of *Fn1* transcriptional variants in MuPαC whose structure is shown in A. The three most highly expressed variants (V1-3) are marked. *C*: Expression (FPKM) levels of total *Fn1* mRNAs in colonic Mu, MuPαC, SM, ICC, SMC, and SMPαC. *D*: c-Fos and c-Jun binding DNA sequence within two peaks (I-1 and I-41) of c-Jun biding sites in A. Three binding sites of c-Fos and c-Jun binding, CATAGTCAT, ATGACGTCAT, and CCAAGTCAG are found at the peak of intron 1 (I-1) from PROMO while one binding site TTGACTCTT is found at intron 41 (I-41).

Using data obtained from our previous smooth muscle transcriptome studies, we built a smooth muscle genome browser utilizing transcriptomes from jejunal and colonic SMC [9], ICC [10], and SMPαC [5] using the UCSC genome browser (UCSC Smooth Muscle Genome Browser; SMGB) [7]. We have now updated the browser with the colonic MuPαC and Mu transcriptome data found in this study. The SMGB now contains the transcriptomic data from colonic SM, SMC, ICC, SMPαC, Mu, and MuPαC along with jejunal SM, SMC, ICC, and SMPαC [6]. This SMGB (found at: https://med.unr.edu/physio/transcriptome) provides not only the genomic map of each splice variant (promoter region, exons, and introns) for all genes expressed in MuPαC, SMPαC, SMC and ICC, but it also allows for analysis of our transcriptome data using the gene expression and regulation data from ENCODE [13] that is available in the database. For example, the genomic structure of *Fn1*, identified as a new marker for MuPαC in this study, is shown in Fig 9A. The *Fn1* gene consists of 46 exons, which are transcribed into 11 different variants in MuPαC (PaC Mu Colon) (Fig 9A). Alternative transcription start sites can be found at E1 (V1), E9 (V2), and E18 (V3). There are also 6 exons (E25, E33, E34, E40, E42, and E44), that are alternatively spliced. Expression levels for each variant can be found in Fig 9B with results showing that V1 (TCONS_00006336: 8,020 bp) is the most highly expressed variant followed by V2 (TCONS_00005374: 6,479 bp), and V3 (TCONS_00001747: 4,954 bp). *Fn1* is expressed at a high level in colonic MuPαC, confirming a low level in the whole tissue Mu, but it is not expressed in colonic SMC, ICC, and SMPαC (Fig 9C), suggesting a mucosa-specific expression of *Fn1*. Through further structural exploration of *Fn1*, a CpG island was found around the promoter, first exon and intron where RNA polymerase 2 was previously found to bind in embryonic fibroblasts (Fig 9A). A region hypersensitive to DNase 1 in NIH3T3 and adult fibroblasts is also found within the same area. These data suggest that *Fn1* is expressed in both embryonic and adult fibroblasts, as well as NIH3T3 cells (fibroblast cell line). In addition, there are two c-Jun binding sites in CH12 cells (lymphoma cell line), found at intron 1 (I-1) and intron 41 (I-41) within the gene (Fig 9A). The two corresponding regions of the c-Jun binging site, 361 bp (chr1: 71698290–71698650) within I-1 and 398 bp (chr1:71640784–71641181) within I-41, were located on the SMGB, and the two DNA sequences were analyzed for the presence of c-Fos and c-Jun binding sites in the transcriptional regulatory element search database, “PROMO” [14]. This search identified three binding sequences, CATAGTCAT, ATGACGTCAT, and CCAAGTCAG at I-1 of *Fn1* gene and one TTGACTCTT at I-41, for both c-Fos and c-Jun (Fig 9D), suggesting the two transcription factors with the highest expression, FOS and JUN (Fig 4D), may transcriptionally regulate *Fn1* via these binding sites. Among 11 *Fn1* variants, V1 (TCONS_00006336), V2 (TCONS_00005374) and V3 (TCONS_00001748) are three major transcripts expressed in MuPαC (Fig 9B). Finally, *Fn1* gene expression was examined in the colonic tissues and cells in the SMGB. The gene expression was restricted to MuPαC, which was confirmed by the expression in Mu and little to no expression in SMC, ICC, SMPαC, and SM (Fig 9C).

## Discussion

In this study, we analyzed the transcriptome obtained from colonic MuPαC and identified signatures of genes including three new MuPαC-specific markers, *Adamdec1*, *Fn1*, and *Col6a4*. Furthermore, we added the transcriptomic data to our Smooth Muscle Genome Browser [9] that already contains transcriptomic data from colonic and jejunal SMC [9], ICC [10], and SMPαC [5]. The browser offers a comprehensive reference for genes expressed not only in colonic MuPαC and Mu, but also colonic and jejunal SMPαC, SMC, and ICC as well as SM.

MuPαC were identified in the colonic mucosa as a unique cellular population that is distinct from subepithelial myofibroblasts [3]. MuPαC and subepithelial myofibroblasts are located in the same anatomical regions and are closely associated underneath epithelial cells [3, 15, 16]. Several markers including PDGFRA, ACTA2, MYH11, DES, and VIM can distinguish the two populations: subepithelial MuPαC (PDGFRA^+^, DES^-^, ACTA2^low^, MYH11^low^, and VIM^low^) and subepithelial myofibroblasts (PDGFRA^-^, DES^+^, ACTA2^high^, MYH11^high^, and VIM^high^) [3, 15]. However, these markers still have overlap between the two cell types at varying levels [3]. Our transcriptome data from colonic MuPαC show a moderate to moderately high expression of of *Acta2* (FPKM: 376) and *Myh11* transcripts (FPKM: 30) in MuPαC (S3 Table). The *Acta2* and *Myh11* gene expression detected in our MuPαC transcriptome data is unlikely due to SMC contamination due to the observation that *Des* is not, or negligibly, detected in the SMC (FPKM: 2) (S3 Table) agreeing with previous findings [3]. This suggests that subepithelial myofibroblasts may be a sub-population of MuPαC.

MuPαC have at least two subpopulations, PDGFRA^high^ (P1: near the apical area of the lamina propria) and PDGFRA^low^ (P2: around the cryptic nadir) (Figs 1A and 2B). PDGFRA^high^ cells are expressed in telocytes/SEMFs (subepithelial myofibroblasts) in the villus and have a role in cell-to-cell communication [16, 17]. *Foxl1*, *Pdgfra*, *Gli1*, *CD34*, and *Cspg4* are ascribed as molecular markers [17, 18]; however, our transcriptome data from colonic MuPαC show high expression of *Pdgfra* (FPKM: 228), while other telocytes/SEMFs markers had low expression [*Foxl1* (FPKM: 32), *Gli1* (FPKM: 44), *CD34* (FPKM: 20) and *Cspg4* (FPKM: 14)] in MuPαC (S4 Table). This suggests that telocytes/SEMFs may be a sub-population of MuPαC. PDGFRA^low^ cells may also express smooth muscle genes. In fact, SMC and PαC are derived from the same mesenchymal precursor cells [19]. Recently, Roulis *et al*., reported the identities of the mesenchymal cell population, which also expresses the PDGFRα^+^ cell marker. Mesenchymal cells have four different fibroblast populations, all populations express *Pdgfra* [20]. In addition, PαC transdifferentiate into SMC in embryonic smooth muscle cells [19, 21], while SMC have the ability to become PDGFRA^low^ cells in response to intestinal injury and under cell culture conditions [11]. These phenotypic overlaps make it hard to identify definitive markers for MuPαC over subepithelial myofibroblasts. The three newly identified MuPαC markers, *Col3a1*, *Adamdec1* and *Col1a2*, are more highly expressed in MuPαC than SMPαC (Fig 2A), suggesting they are better markers for MuPαC than PDGFRA alone. Further studies should explore if these new markers can distinguish MuPαC over subepithelial myofibroblasts.

Through transcriptomic analysis, we compared genes of interest between 1) MuPαC and Mu or 2) MuPαC and SMPαC throughout this manuscript. We were able to identify the top thirty genes that are enriched and selectively expressed in MuPαC over Mu. Next, we identified the top thirty genes that are enriched and selectively expressed in MuPαC over SMPαC. With limited space, we discussed only the most or second most expressed genes in each functional gene category. As it pertains to growth factors, we found *Cxcl12* and *Ogn* are the most highly expressed genes in MuPαC. *Cxcl12* encodes for the C-X-C Motif Chemokine Ligand 12 which functions as a ligand for the G-protein couple receptor 4 (CXCL12). This ligand regulates embryogenesis [22], stem cell homeostasis [23], immune surveillance [22], tissue regeneration [22], inflammation [24], and tumorigenesis [25]. Another growth factor found in MuPαC, *Ogn*, encodes osteoglycin which also regulates fibrosis [26], immune response [27], inflammation [28], and colon cancer [29]. A family of growth factors, the *Bmp* genes (*Bmp*7, 5, 3, 4, 1), are within the top ten genes of growth factors that are highly expressed in MuPαC. BMPs (bone morphogenetic proteins) belong to the transforming growth factor-β (TGFβ) superfamily. BMP7 is mostly expression in tumor including colon cancer, and it is regulated of cell proliferation [30]. Recent studies have shown that BMPs play an important role in regulating the immune response to infection, inflammation [31], and cancer [30, 32]. In regard to the transcription factors expressed in MuPαC, we found *Fos* and *Jun* to be the most highly expressed genes in MuPαC. FOS and JUN form a heterodimer, forming the transcription factor AP1 (Activator Protein 1) that regulates the expression of genes involved in cell proliferation, differentiation, and apoptosis [33]. JUN is essential for fibroblast proliferation [34], and TGFβ stimulated cell proliferation via FOS [35], suggesting that AP1 could regulate the proliferation of MuPαC via BMPs.

The most abundantly expressed gene group in MuPαC are those related to extracellular activity (Fig 1E). Not only are they highly expressed, but they are also the largest gene group (600 genes) represented in MuPαC (S10 Table). Collagen types, 3, 1, and 6 are among the thirty most highly expressed genes in MuPαC (Fig 6D). Collagen is amongst the most abundant protein made in mammals, representing 25–30% of all proteins produced. Twenty collagen isoform genes, including type 1, 3, 4, 5, 6, 8, 12, 14, 15, 16, 18, 20, 24, and 27 are expressed in MuPαC. Most of these isoforms are also abundantly expressed in SMPαC with *Col6a4* being negligibly expressed in S SMPαC, thus, we have identified *Col6a4* as a MuPαC-specific marker (Fig 2). The transcriptome data from isolated cells confirm that this gene is expressed in MuPαC, but insignificantly expressed in SMPαC (Fig 3). The mucosa specific expression is consistent with the gene expression level in the transcriptome data found in both mucosa (Mu) and smooth muscle tissue (SM). Unfortunately, a COL6A4-specific antibody is not currently available. Therefore, we used an anti-collagen type 6 antibody which detects all COL6A isoforms, COL6A1-6. The immunohistochemical data in Fig 3 shows the protein is abundantly found within mucosa restricted to MuPαC as well as smooth muscle tissue mainly in SMPαC. The transcriptome data show that *Col6a1*, *Col6a2*, and *Col6a3* had more expression in both MuPαC and SMPαC compared to SMC and ICC (S4 Table), suggesting that COL6A1-3 are mainly expressed in colonic PαC (SMPαC and MuPαC). The *Col6a4* gene encodes for COL6A4 protein in mice, but is only a pseudogene in humans, which limits direct human application of this gene. Nevertheless, we demonstrated that *Col6a4* gene products (mRNAs and protein) in mice can be used for a selective marker for MuPαC. In addition, the gene locus may be a useful target to generate MuPαC-restricted mouse lines.

In total, we were able to identify 15,777 genes expressed into 51,282 unique transcripts within isolated MuPαC. This valuable gene expression data was added to our “Smooth Muscle Genome Browser” [6] that contains the transcriptome data from colonic and jejunal SMC [9], ICC [10], and SMPαC [5]. This browser provides comprehensive genetic information in designated cell populations in both the colon and jejunum. In addition, through the browser, users can access the gene expression and regulation data (ENCODE) in the genome database [13] that allows for the study of genetic and epigenetic regulation of genes expressed within specific cell populations. However, there are some limitations to using the Smooth Muscle Genome Browser. For example, it cannot display an expression level and cDNA of an individual gene or transcriptional variant expressed in these specific cell populations. To rectify this issue, we built another browser: “Smooth Muscle Transcriptome Browser” [6]. This additional browser offers genetic references and expression profiles (expression levels, cDNAs, and encoded protein) of all transcripts expressed in individual cell populations and their associated tissues. Both browsers are available online, hosted by the University of Nevada, Reno at https://med.unr.edu/physio/transcriptome. These two browsers provide genome-wide genetic references and expression levels that bring advanced levels of insight into genetic structure, expression profile, and the isoforms of each gene expressed in intestinal cell groups and muscularis and mucosal tissue. We anticipate that these two browsers will greatly improve studies on GI smooth muscle biology and physiology.

In summary, we have analyzed the transcriptome of colonic MuPαC and identified signature genes including new selective markers relevant to cell identity and functionality. This transcriptome data was added to our Smooth Muscle Genome Browser and Smooth Muscle Transcriptome Browser that both offer vital genetic references for PDGFRα^+^ cells that can aid further functional studies in intestinal diseases and physiology.

## Supporting information

S1 File (DOCX)

S1 Fig Non-selective expression of PLUA, PROCR, BMP7, SEMA3F and PCSK6 in colonic MuPαC. Vertical and horizontal cross-sections (CS) images are indicated. Scale bars are 50 μm.
(TIF)

S2 Fig Validation of expression levels of MuPαC-selective genes. (**a-f**) Expression levels of MuPαC-selective genes in MuPaC, SMPαC, ICC, SMC, colonic Mu and SM tissue measured by qPCR. *A*: *Col3a1* and *Adamdec1* in Fig 2. *B* and *C*: *Bmp7*, *Bmp5*, *Ogm*, *Foxf2*, *Tbx3* and *Tbx2* in Fig 4. *D* and *E*: *Wnt4*, *Wnt5a*, *Pdgfra*, *Nlrp6* and *Agt* in Fig 5. *F*: *Gja1*, *Gjb1* and *Gjb3* in Fig 6. n = 5–6 per groups. * *p* ≤ 0.05 and ** *p* ≤ 0.01, versus MuPαC.
(TIF)

S3 Fig Validation of expression levels of MuPαC-selective genes. *A*-*D*: Expression levels of MuPαC-selective genes in MuPαC, SMPαC, ICC, SMC, colonic Mu and SM tissue measured by qPCR. *A* and *B*: *Dmp1*, *Cxc9*, *Fam3b*, *Masp1* and *Penk1* in Fig 7. *C* and *D*: *Axl*, *Rps6ka1*, *Vegfa*, *Dusp10* and *Ptpn13* in Fig 8. n = 5–6 per groups. * *p* ≤ 0.05 and ** *p* ≤ 0.01, versus MuPαC.
(TIF)

S4 Fig (PDF)

S1 Table Oligonucleotides used in this study. (XLSX)

S2 Table List of transcriptional variants expressed in colonic Mu and MuPαC. (XLSX)

S3 Table List of genes expressed in colonic Mu and MuPαC. (XLSX)

S4 Table List of genes highly and selectively expressed in colonic Mu and MuPαC. (XLSX)

S5 Table List of growth factors expressed in colonic Mu and MuPαC. (XLSX)

S6 Table List of transcription factors expressed in colonic Mu and MuPαC. (XLSX)

S7 Table List of cell signaling genes expressed in colonic Mu and MuPαC. (XLSX)

S8 Table List of receptors and receptor binding proteins expressed in colonic Mu and MuPαC. (XLSX)

S9 Table List of gap junction proteins expressed in colonic Mu and MuPαC. (XLSX)

S10 Table List of extracellular proteins expressed in colonic Mu and MuPαC. (XLSX)

S11 Table List of cytokines expressed in colonic Mu and MuPαC. (XLSX)

S12 Table List of peptidase expressed in colonic Mu and MuPαC. (XLSX)

S13 Table List of protein kinase activity expressed in colonic Mu and MuPαC. (XLSX)

S14 Table List of phosphatases expressed in colonic Mu and MuPαC. (XLSX)
