# Supplementary figures and images for: Transcriptome profiling of subepithelial PDGFRα cells in colonic mucosa reveals several cell-selective markers
Source: PLoS One. 2022 May 13;17(5):e0261743. doi: 10.1371/journal.pone.0261743 (PMC9106222; doi:10.1371/journal.pone.0261743)

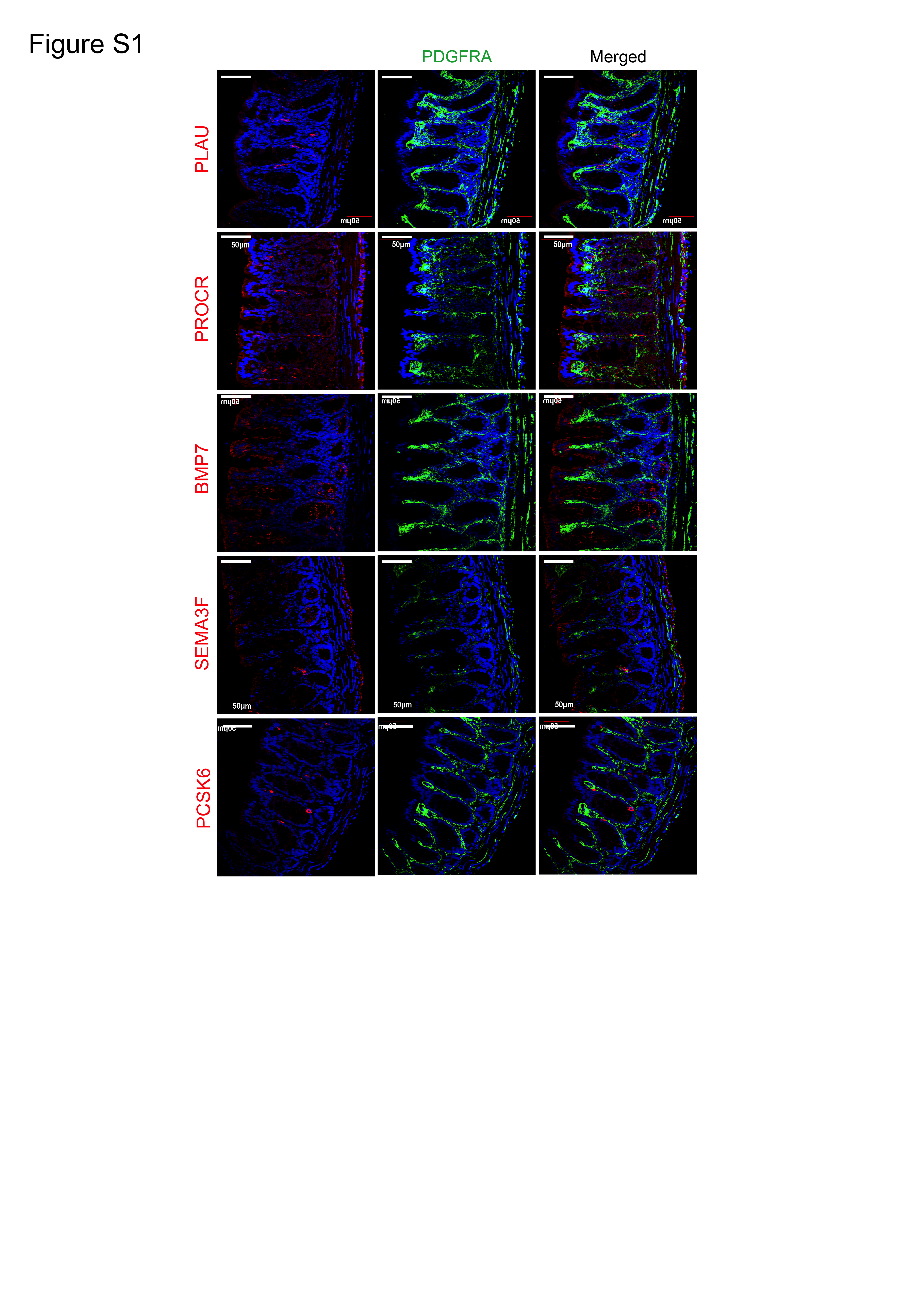

Supplement: S1 Fig — Vertical and horizontal cross-sections (CS) images are indicated. Scale bars are 50 μm. (TIF) [file pone.0261743.s002.tif]

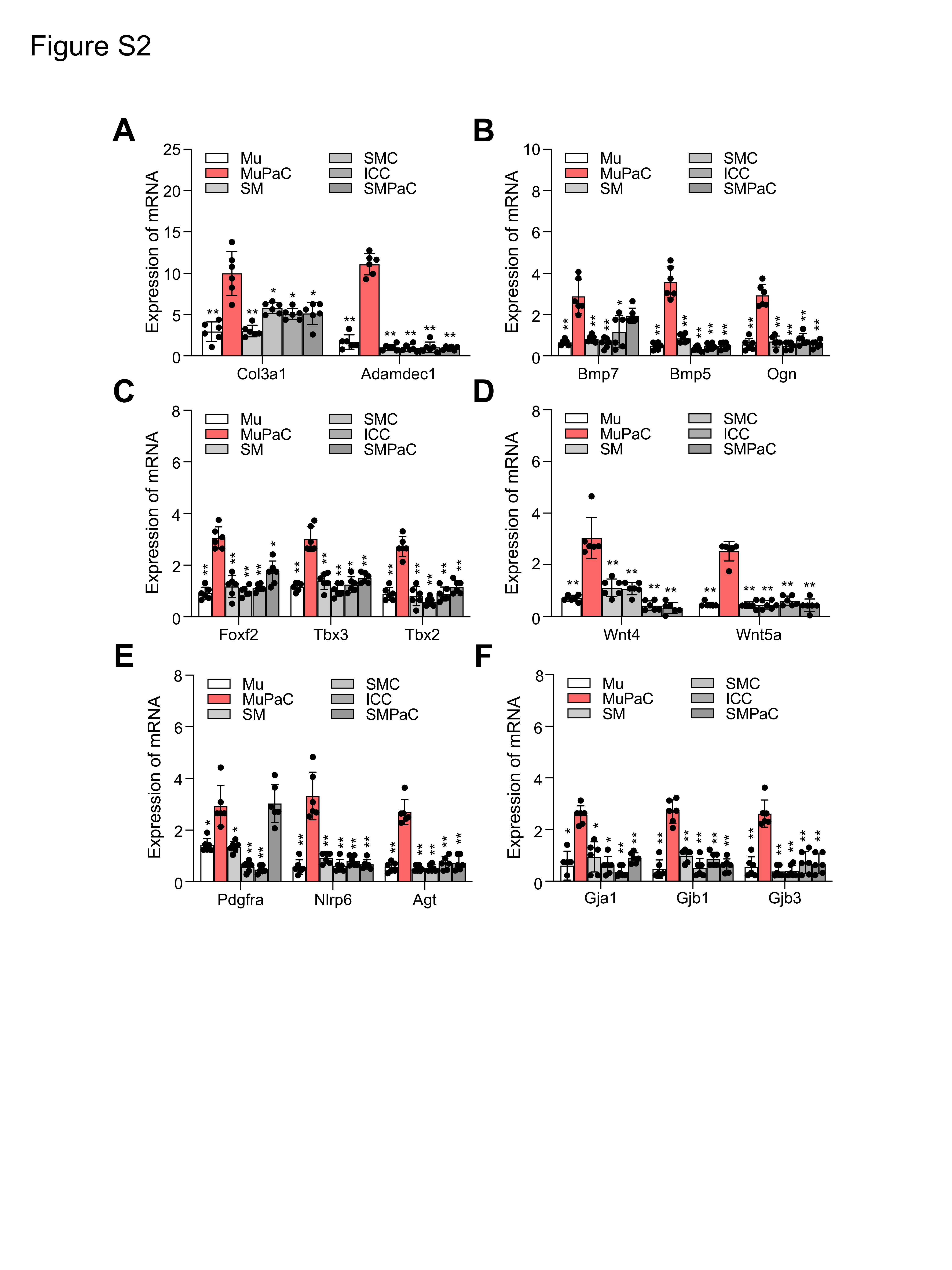

Supplement: S2 Fig — (a-f) Expression levels of MuPαC-selective genes in MuPaC, SMPαC, ICC, SMC, colonic Mu and SM tissue measured by qPCR. A: Col3a1 and Adamdec1 in Fig 2. B and C: Bmp7, Bmp5, Ogm, Foxf2, Tbx3 and Tbx2 in Fig 4. D and E: Wnt4, Wnt5a, Pdgfra, Nlrp6 and Agt in Fig 5. F: Gja1, Gjb1 and Gjb3 in Fig 6. n = 5–6 per groups. * p ≤ 0.05 and ** p ≤ 0.01, versus MuPαC. (TIF) [file pone.0261743.s003.tif]

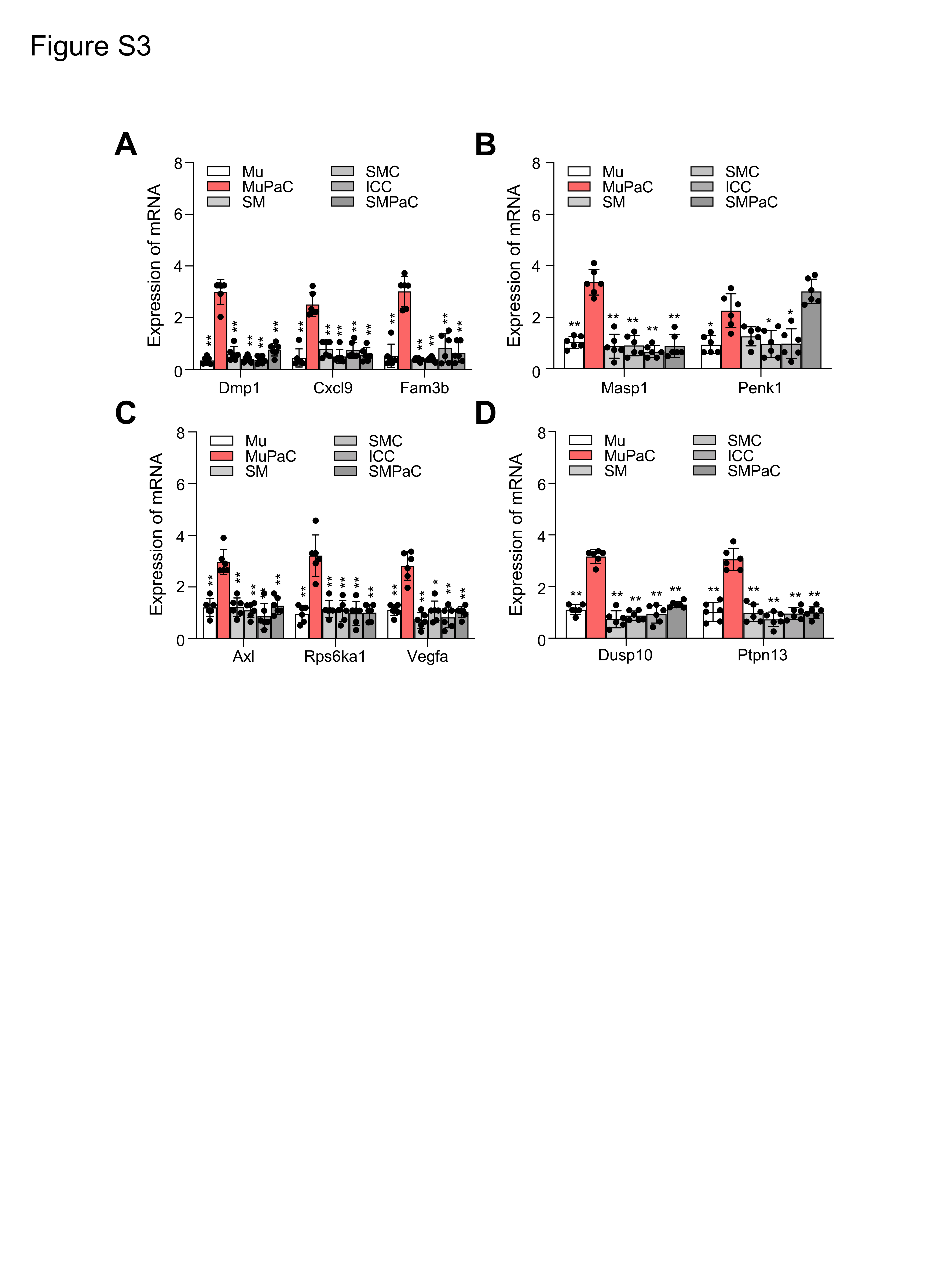

Supplement: S3 Fig — A-D: Expression levels of MuPαC-selective genes in MuPαC, SMPαC, ICC, SMC, colonic Mu and SM tissue measured by qPCR. A and B: Dmp1, Cxc9, Fam3b, Masp1 and Penk1 in Fig 7. C and D: Axl, Rps6ka1, Vegfa, Dusp10 and Ptpn13 in Fig 8. n = 5–6 per groups. * p ≤ 0.05 and ** p ≤ 0.01, versus MuPαC. (TIF) [file pone.0261743.s004.tif]

Fig 3c\_Adamdec1

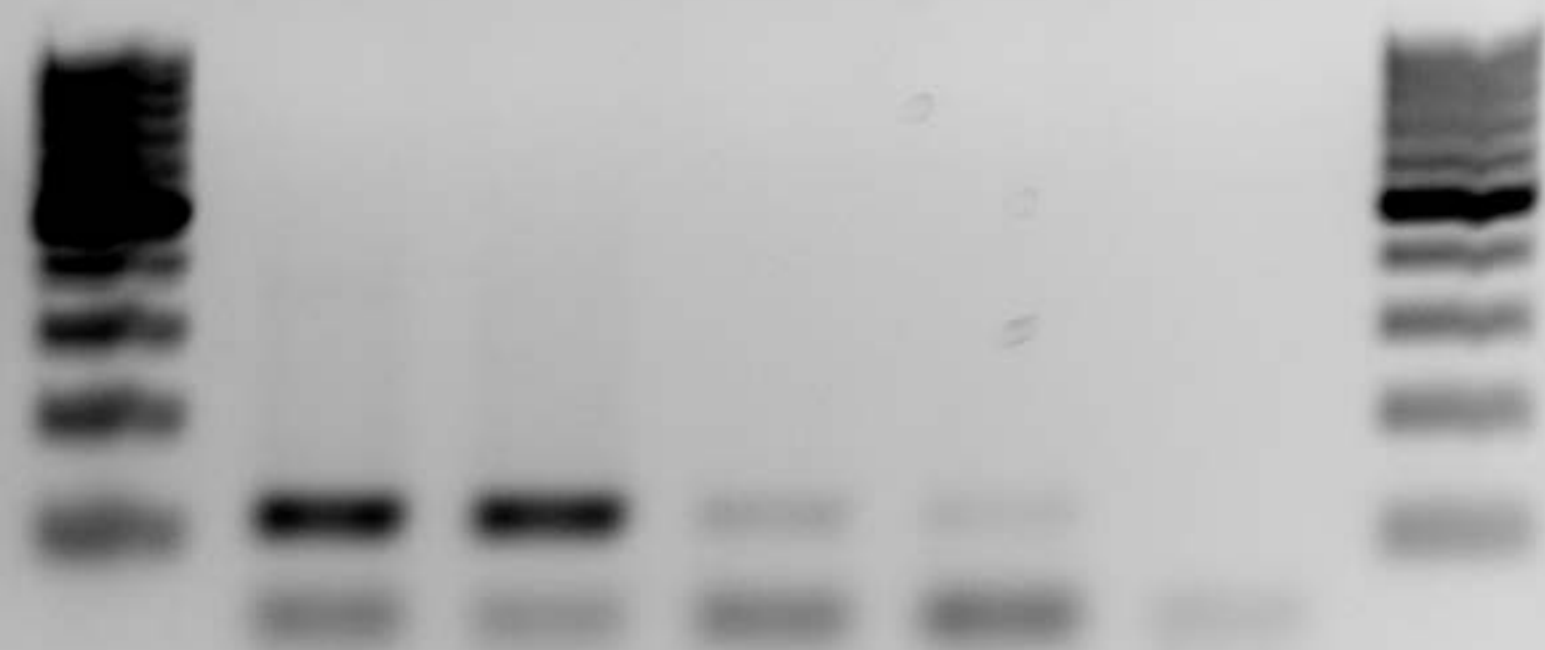

Fig 3c\_Fn1

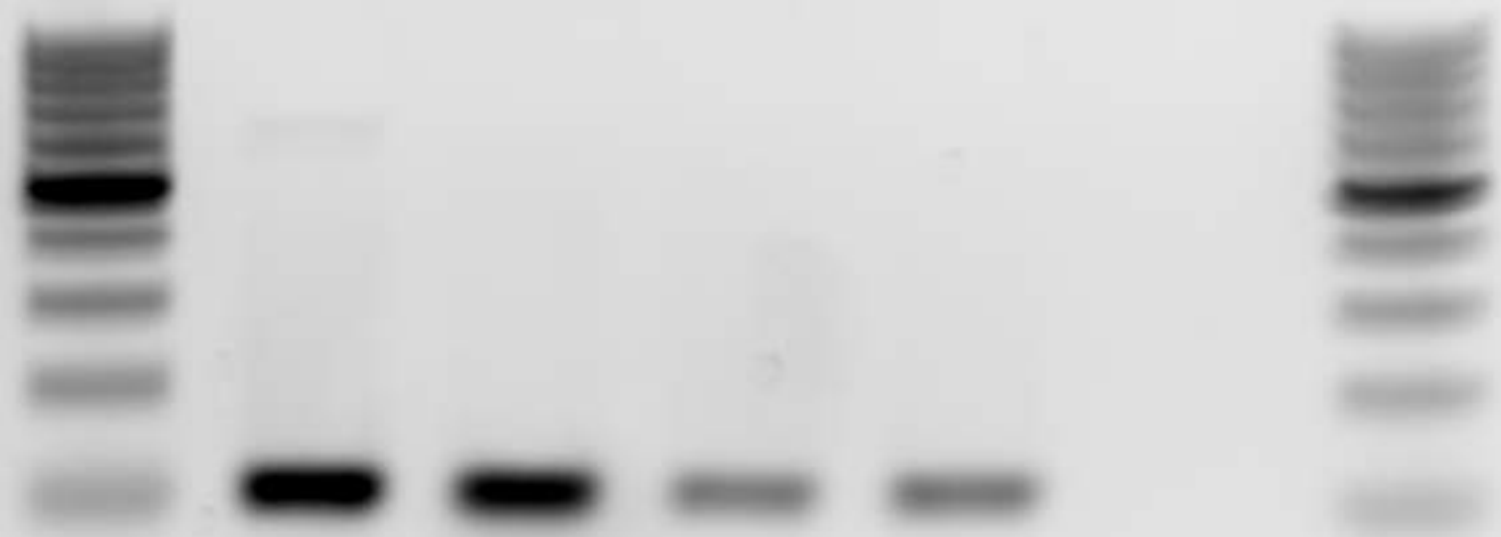

Fig 3c\_Col6a1

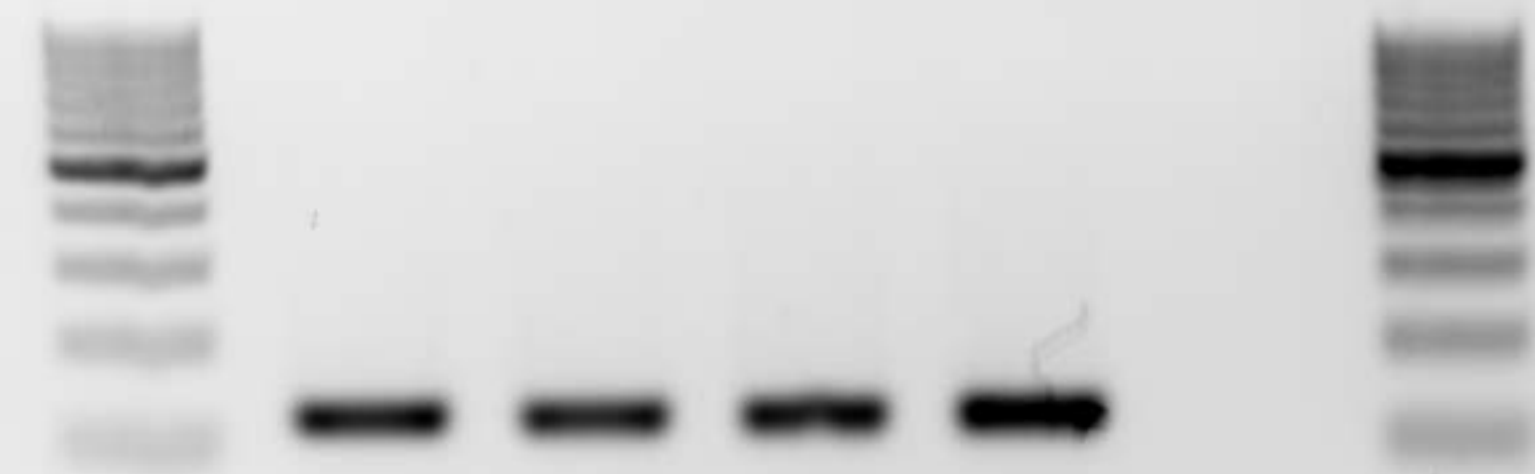

Fig 3c\_Col6a2

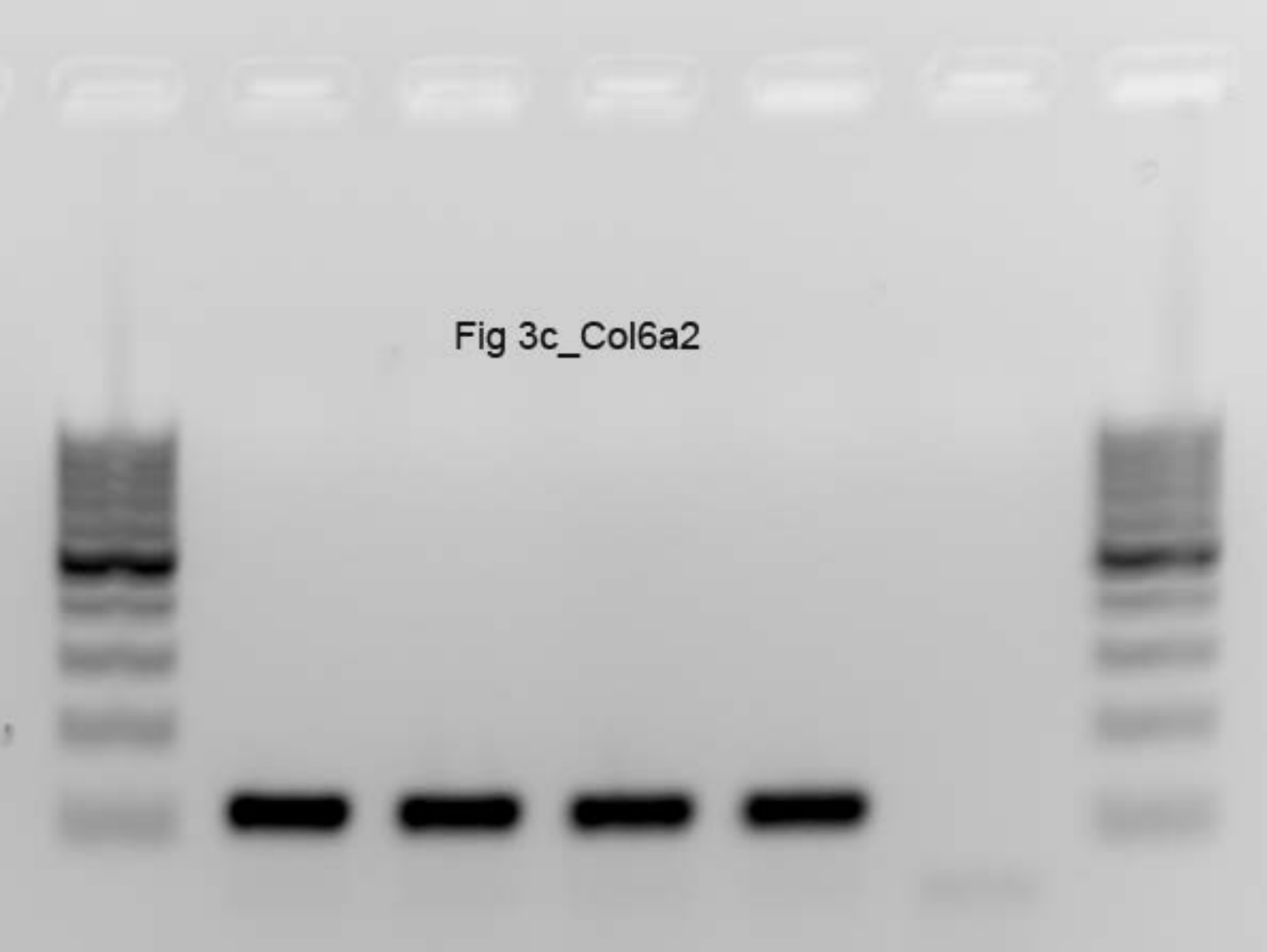

Fig 3c\_Col6a3

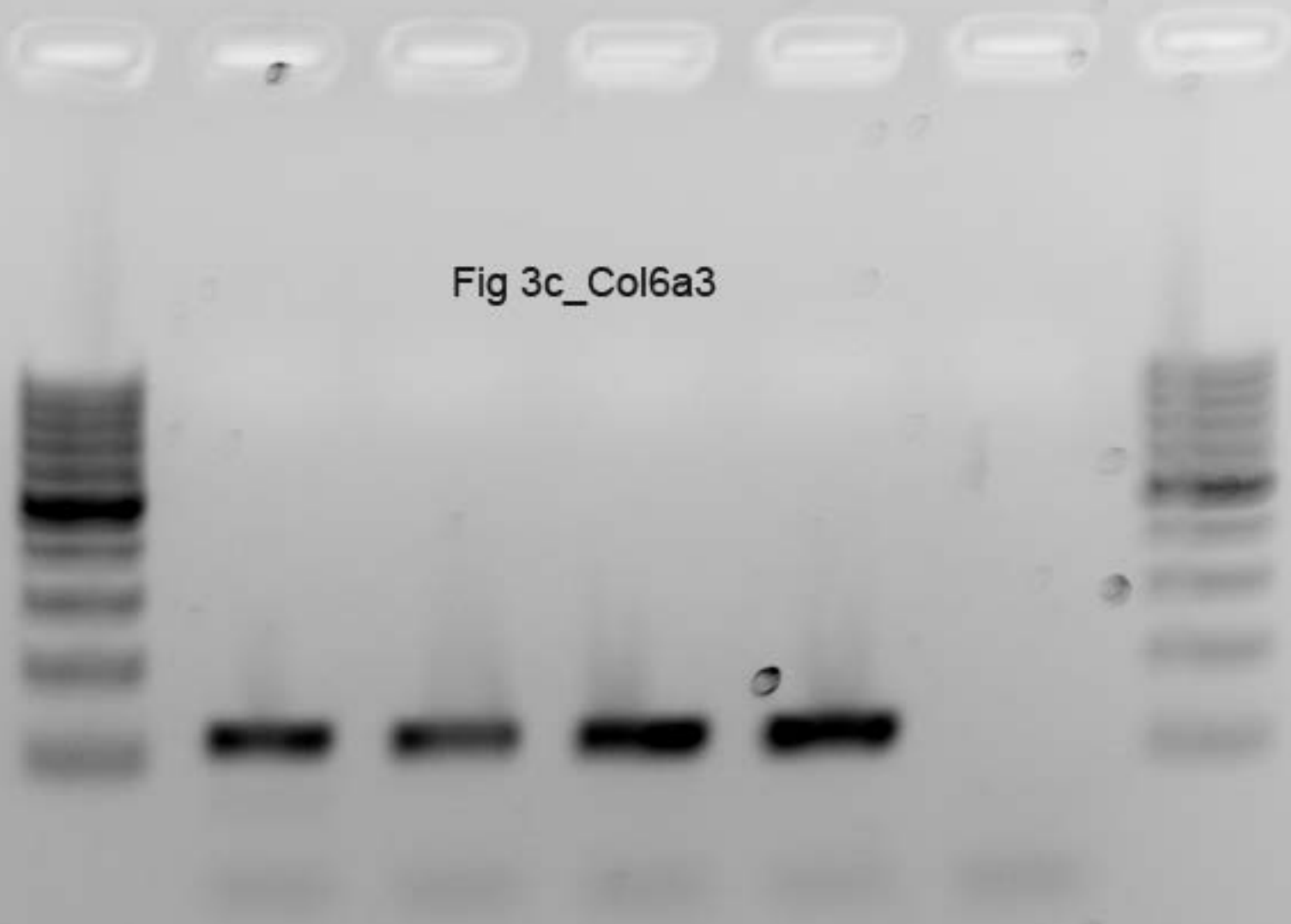

Fig 3c\_Col6a4

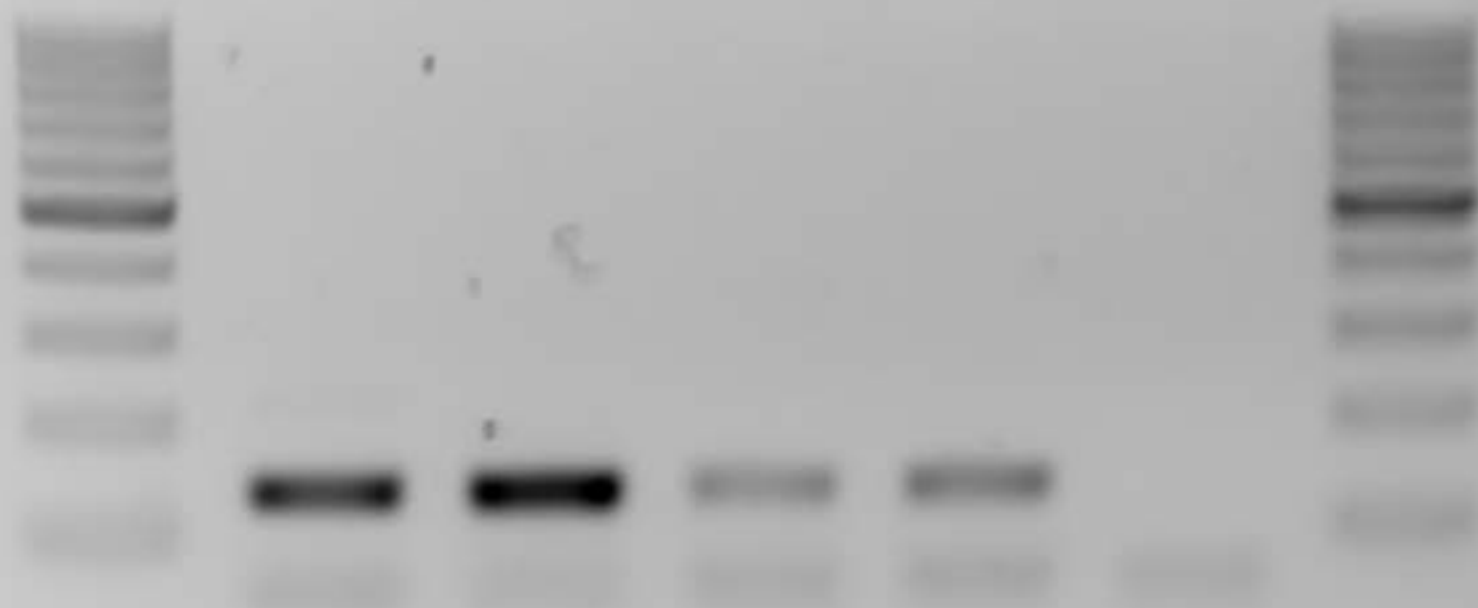

Fig 3c\_Col6a5

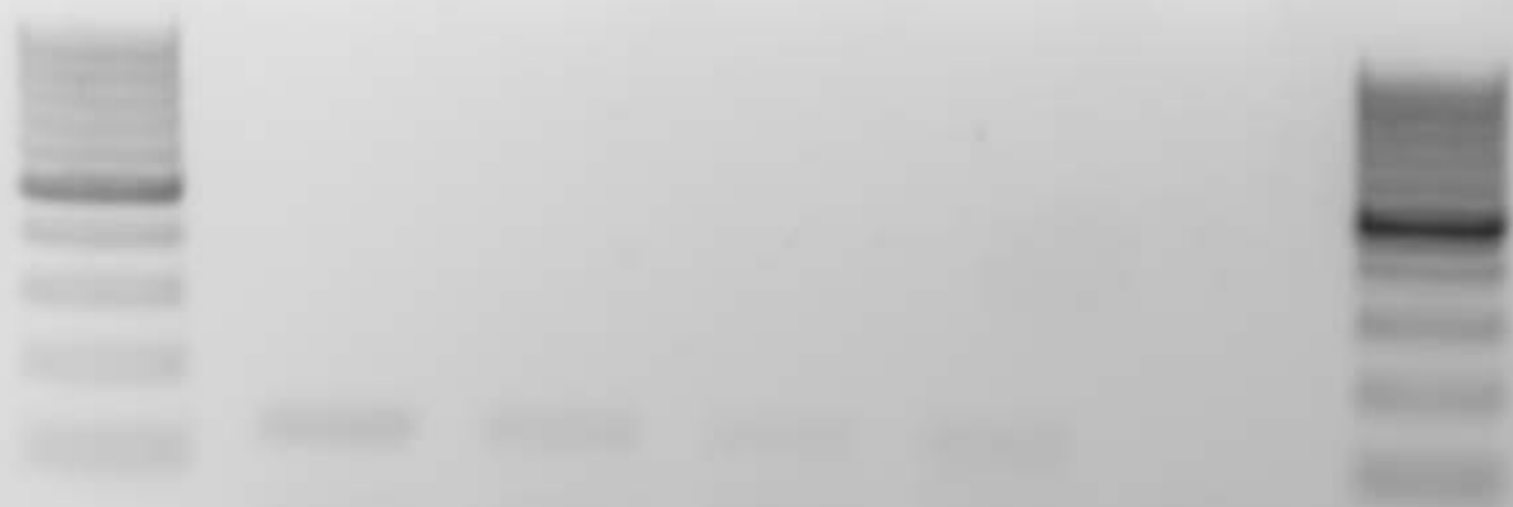

Fig 3c\_Gapdh

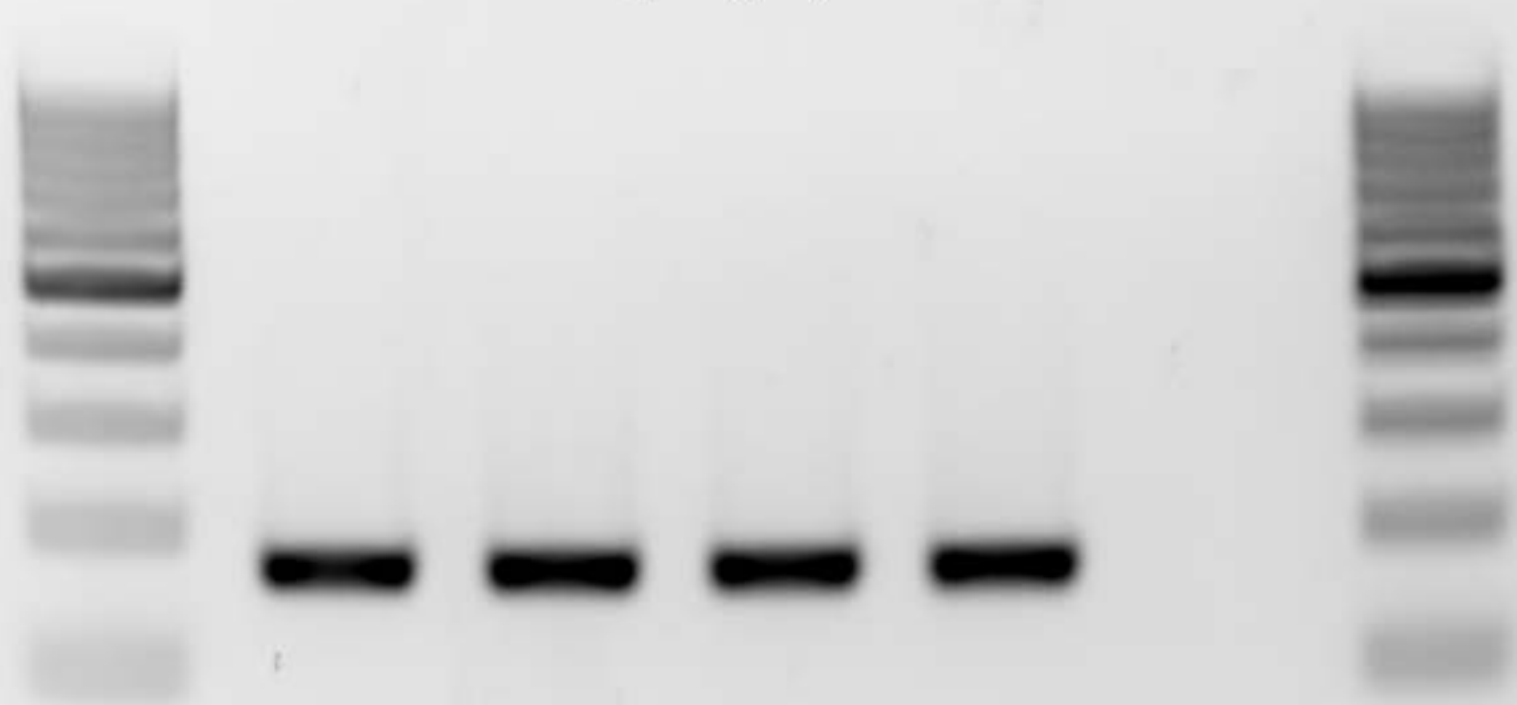

Supplement: S4 Fig — (PDF) [file pone.0261743.s005.pdf]
